# Neuronal ribosomes exhibit dynamic and context-dependent exchange of ribosomal proteins

**DOI:** 10.1038/s41467-021-26365-x

**Published:** 2021-10-21

**Authors:** Claudia M. Fusco, Kristina Desch, Aline R. Dörrbaum, Mantian Wang, Anja Staab, Ivy C. W. Chan, Eleanor Vail, Veronica Villeri, Julian D. Langer, Erin M. Schuman

**Affiliations:** 1 Max Planck Institute for Brain Research, Frankfurt, Germany; 2 Max Planck Institute for Biophysics, Frankfurt, Germany; 3 Present Address: MOS, Center for Mass Spectrometry and Optical Spectroscopy, Mannheim, Germany; 4 Present Address: Institute of Molecular and Clinical Ophthalmology, Basel, Switzerland; 5 Present Address: German Center for Neurodegenerative Diseases, Bonn, Germany; 6 Present Address: Department of Neuroscience, University of Bordeaux, Bordeaux, France

**Keywords:** Cell polarity, Molecular neuroscience

## Abstract

Owing to their morphological complexity and dense network connections, neurons modify their proteomes locally, using mRNAs and ribosomes present in the neuropil (tissue enriched for dendrites and axons). Although ribosome biogenesis largely takes place in the nucleus and perinuclear region, neuronal ribosomal protein (RP) mRNAs have been frequently detected remotely, in dendrites and axons. Here, using imaging and ribosome profiling, we directly detected the RP mRNAs and their translation in the neuropil. Combining brief metabolic labeling with mass spectrometry, we found that a group of RPs rapidly associated with translating ribosomes in the cytoplasm and that this incorporation was independent of canonical ribosome biogenesis. Moreover, the incorporation probability of some RPs was regulated by location (neurites vs. cell bodies) and changes in the cellular environment (following oxidative stress). Our results suggest new mechanisms for the local activation, repair and/or specialization of the translational machinery within neuronal processes, potentially allowing neuronal synapses a rapid means to regulate local protein synthesis.

## Abstract (web summary)

Ribosomes, assembled in the nucleus and perinuclear region, are thought to remain invariant throughout their lifetime. Here the authors document the dynamic association or exchange of ribosomal proteins within mature ribosomes of neuronal cells.

## Introduction

Neurons use the translation of distally localized messenger RNAs (mRNAs) for synapse formation, axon growth, and synaptic plasticity^1^. Although ribosomes have been detected in dendrites^2^ and axons^3,4^, little is known about ribosome biogenesis and homeostasis in neurons. Thus far, studies from yeast to human have revealed a striking conservation of the basic ribosome structure^5^. Eukaryotic ribosomes are composed of a small and a large subunit comprising ~79 proteins (ribosomal proteins, RPs) and 4 rRNA species. In eukaryotes, ribosomal components (including most RPs and rRNA) are initially co-assembled in the nucleolus. The nearly mature ribosome is then exported to the cytoplasm where a few RPs associate to complete the maturation process^6^. The majority of RPs are thought to exhibit a stable, “life-long” incorporation with their associated subunits, and, at the end of their life-cycle, they are thought to undergo concerted degradation^7^.

Recent data, however, have suggested that ribosomes may be less static than the above picture suggests^8–10^. Proteomic data, for example, have reported ribosomes containing individual ribosomal proteins at different stoichiometries with unique translational properties^11,12^. As ribosome biogenesis is believed to require the regulated incorporation of all ribosomal proteins, it remains unclear how heterogeneous ribosomes are formed. Additionally, many transcriptomics studies have detected RP mRNAs remote from the nucleus, including in distal neuronal processes, raising questions about current ideas of ribosome biogenesis^4,13–32^. Surprisingly, the cytosolic incorporation of some RPs was recently reported in the developing axons of *Xenopus* retinal ganglion cells^8^. Altogether, the above data suggest that the protein composition of ribosomes might not be fixed after biogenesis, but rather remain susceptible to dynamic association or exchange of RPs with mature ribosomes.

To address this possibility in rodent neurons, we first used high-resolution fluorescence in situ hybridization (FISH) to directly detect a large population of RP mRNAs in neuronal cell bodies and dendrites. Using ribosome footprinting and metabolic-labeling approaches we observed the active translation of RP mRNAs in the neuropil. The dendritic synthesis of RPs, remote from the perinuclear region, prompted us to investigate the dynamics of RP association with mature neuronal ribosomes. We used very brief metabolic labeling (dynamic SILAC) combined with parallel-reaction monitoring mass spectrometry to evaluate selectively the abundance of individual “new” and “old” RP peptides within ribosomes. We identified a population of 12 nascent RPs (“exchangers”) that rapidly incorporate into mature pre-existing ribosomes. Using compartmentalized chambers, we observed the nucleus-independent incorporation of RPs in both somata and isolated neuronal processes. Moreover, we found that the incorporation probability of some RPs was regulated by the subcellular compartment (neurites vs. cell bodies) and by changes in the physiological state (following oxidative stress). Taken together, these data suggest that neurons can dynamically regulate RPs incorporation into ribosomes in space and time.

## Results

### RP mRNA localization and translation in dendrites

**Fig. 1:**
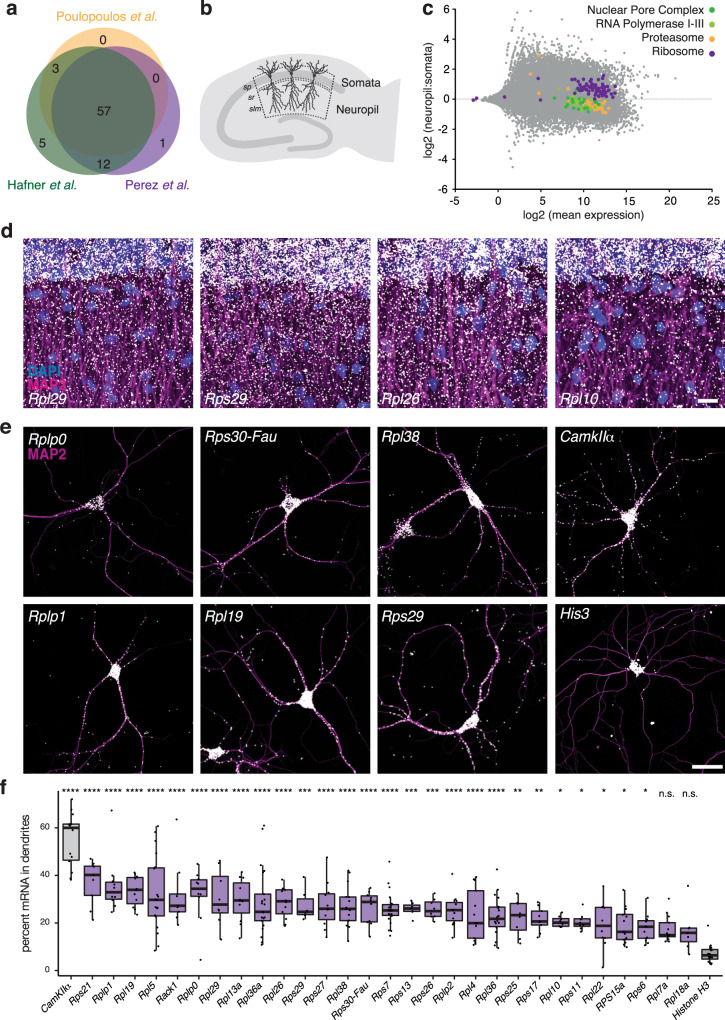
RP mRNAs are present in hippocampal dendrites. **a** Overlap of RP mRNAs detected in neuronal processes from three different studies: dendritic arbors (ref. ^26^, in purple), purified synaptosomes (ref. ^4^, in green), developing axons (ref. ^27^, in yellow) of rodent neurons. See also Supplementary Data 1 for additional studies and data. **b** Schematic representation of a hippocampal slice. Three layers in area CA1 are shown: somata (*stratum pyramidale*, rich in cell bodies) and neuropil (*strata radiatum et lacunosum-moleculare*, rich in axons and dendrites^77^. **c** MA plot depicting the expression of mRNAs from the somata and neuropil of hippocampal slices^14^. Each mRNA is represented by a single data point. The *y*-axis depicts the relative expression in the neuropil or somata and the *x*-axis depicts the mean expression of each mRNA. Unlike mRNAs for other macromolecular complexes (e.g., the proteasome, nuclear pore complex and RNA polymerase I–III), mRNAs coding for ribosomal proteins are consistently enriched in the neuropil. **d** FISH detection of indicated RP mRNAs in dendrites (magenta MAP2, white FISH, blue DAPI) in hippocampal slices. Images are oriented with the somata layer at the top and the dendrites extending below. Scale bar = 50 µm. See Supplementary Fig. 1a, b for additional RPs and analysis. Representative images from *n* ≥ 7 field of views, acquired over ≥2 biologically and technically independent experiments, **e** FISH detection of indicated RP mRNAs as well as CamkIIα and His3 mRNA (magenta MAP2, white FISH) in cultured hippocampal neurons. Scale bar = 50 µm. See Supplementary Fig. 2 for additional RPs. Representative images from n ≥ 6 field of views. **f** Analysis of FISH data shown in Fig. 1e and Supplementary Fig. 2. Percentage of indicated mRNA signal in dendrites over the total detected in single neurons. Transcripts are ranked according to average value. The distribution of each mRNA was compared to the largely somatically localized transcript *His3* (Ordinary one-way ANOVA (p < 0.0001) followed by a Dunnett’s multiple comparison test using *His3* as control group, *p ≤ 0.05, **p ≤ 0.01, ***p ≤ 0.001, ****p ≤ 0.0001). Center of the box plots represents the median, hinges include first and third quartiles, and whiskers extend up to the smallest/largest value included in 1.5-fold the interquartile range (IQR). *n* of cells between 6 and 22 (as indicated by the dot plots).

Advances in transcriptome-wide profiling methods have led to the elucidation of thousands of mRNAs localized to neuronal processes. In addition to many neuronal/synaptic transcripts, the RP mRNAs have been surprisingly detected in many preparations enriched for axons and dendrites (Fig. 1a and Supplementary Data 1; see also refs. ^4,13,14,17–19,22,24,26–32,33^). To evaluate whether the mRNAs for the ribosome are specifically enriched in dendrites and axons, we compared the dendritic enrichment of RP mRNAs to mRNAs that code for proteins in other ubiquitous macromolecular complexes. We used total RNA-seq data^14^, comparing somata-enriched or neuropil fractions of rat hippocampal slices (Fig. 1b) and quantified the neuropil enrichment of mRNAs coding proteins of the ribosome (RPs), proteasome, nuclear pore complex and RNA polymerase I–III (Fig. 1c). We found that only the RP mRNAs exhibited a consistent enrichment in the neuropil, while the mRNAs of all other complexes were mostly enriched in somata. This suggests that the neuropil localization of RP mRNAs is not owing to “background” detection of abundant mRNAs or the presence of contaminants (e.g., glia cells) in the sequenced material.

To assess directly whether the RP mRNAs are localized in dendrites, we performed single molecule fluorescence in situ hybridization (smFISH) for 29 different endogenous RP transcripts in both rat hippocampal slices (Fig. 1d and Supplementary Fig. 1a) and cultured rat hippocampal neurons (Fig. 1e and Supplementary Fig. 2). In hippocampal slices, for each RP transcript evaluated, we detected signal in the somata (*s. pyramidale*) and in the neuropil (*s. radiatum*) at levels similar to those measured by RNAseq (Supplementary Fig. 1b, c). Likewise, in cultured hippocampal neurons, we detected abundant RP mRNAs both in the cell body as well as in the dendrites. To normalize for potential differences in expression level, we quantified the fraction of the total mRNA signal detected in the dendrites of individual neurons. Amongst the 29 RP mRNAs we evaluated, 15% (e.g., Rpl7a) to 40% (e.g., Rps21) of the total mRNA was localized in dendrites (Fig. 1f). For comparison we analyzed the dendritic abundance of a well-studied and abundant dendritic mRNA Ca^2+^-calmodulin-dependent protein kinase, CamKIIα^18,34,35^, and a somatic-enriched mRNA encoding the nuclear protein histone H3-3B^18^. As expected, a high fraction (60%) of CamKIIα mRNAs and a very low fraction (6%) of Histone 3 mRNAs were detected in dendrites. Twenty-seven of the 29 tested RP mRNAs exhibited a distribution in the dendrites significantly greater than that observed for the nuclear protein encoding mRNA Histone H3 (Fig. 1f). Taken together, these data demonstrate that RP mRNAs are localized to the neuropil and dendrites of hippocampal slices and cultured neurons.

**Fig. 2:**
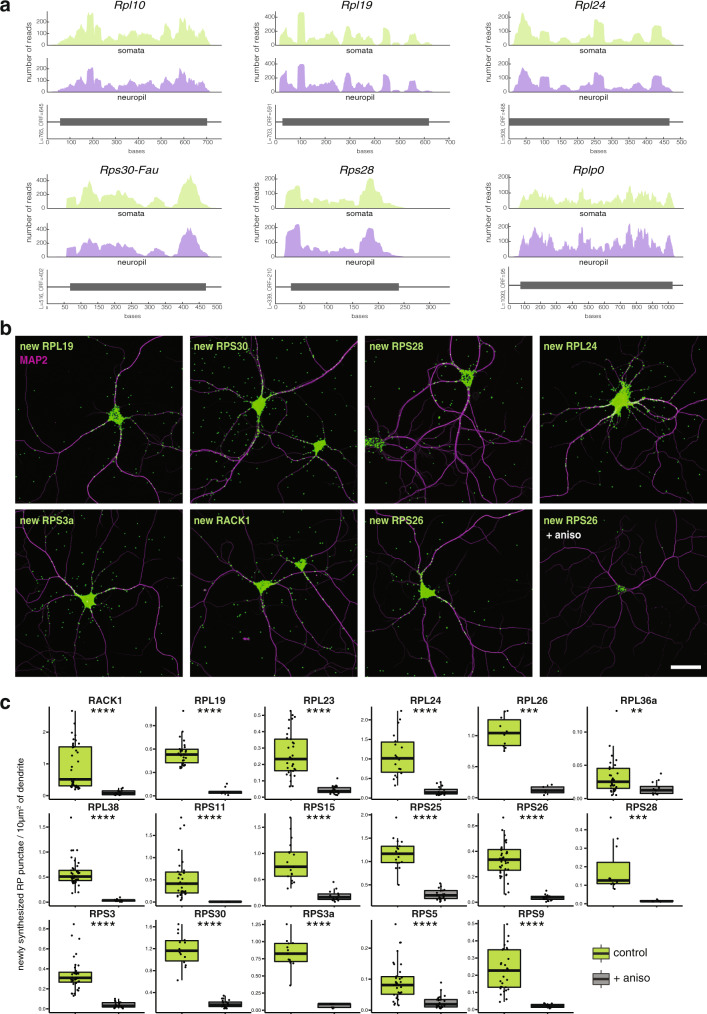
RP mRNAs are translated in hippocampal dendrites. **a** Ribosome footprint coverage of RP mRNAs from the somata and neuropil of hippocampal slices^14^. Shown are the number of reads throughout the open reading frame (gray box) from the somata-enriched fraction (green) or the neuropil-enriched fraction (purple). Ribosome footprint coverage for additional RPs is shown in Supplementary Fig. 3. **b** Detection of nascent RPs (green) in dendrites (magenta for MAP2 immunostaining) of cultured hippocampal neurons using Puro-PLA^37^. Nascent proteins were labeled with puromycin (5 min), in the absence or presence (as indicated) of the protein synthesis inhibitor anisomycin (see methods). Scale bar = 50 µm. Data for additional nascent RP detection are shown in Supplementary Figs. 4b and 5. Representative images from *n* ≥ 7 field of views, acquired over ≥2 biologically and technically independent experiments. **c** Analysis of nascent RP detection shown in Fig. 2b, Supplementary Figs. 4b and 5. Analyzed are the newly synthesized RP punctae per 10 µm^2^ of dendrite, in control (green) or in the presence of protein synthesis inhibitor (gray). Each dot is the quantification from the whole dendritic arbor of one single neuron. Wilcox test, **p ≤ 0.01, ***p ≤ 0.001, ****p ≤ 0.0001. Center of the box plots represents the median, hinges include first and third quartiles, and whiskers extend up to the smallest/largest value included in 1.5-fold the interquartile range (IQR). *n* of cells between 4 and 42 (as indicated by the dot plots), over at least 2 independent experiments.

We next asked whether the dendritically localized RP mRNAs are locally translated into protein. We investigated via ribosome profiling whether RP mRNAs are associated with translating ribosomes in the cell bodies (somata) and/or neuropil of the hippocampus. Using our dataset^14^, we detected ribosome footprints across the coding sequence of each RP transcript measured in the neuropil, a region enriched for axons and dendrites (Fig. 2a and Supplementary Fig. 3). Analysis of the footprint abundance revealed that all RP mRNAs were either equally translated within the two compartments (somata or neuropil) or exhibited significantly enhanced translation in the neuropil (Supplementary Fig. 4a). We note that the translation of RPs has also been detected in mouse retinal ganglion cell axons and cortical synaptoneurosomes using ribosome-bound mRNA sequencing^16,29^. As the somata and neuropil fractions also contain glia and other cell types, we validated the translation of the RPs in situ. We used puromycin proximity ligation assay (Puro-PLA) to visualize newly synthesized proteins-of-interest^36^ and observed nascent signal within the dendrites for all 17 RPs examined with just 5 min of metabolic labeling (Fig. 2b and Supplementary Fig. 5). As one noteworthy example, almost half of the total RPL19 signal was observed in the dendrites (Supplementary Fig. 4c). As expected, the addition of a protein synthesis inhibitor significantly inhibited the dendritic nascent protein signal of all RPs (Fig. 2b, c and Supplementary Figs. 4b and 5). Moreover, the nascent dendritic protein signal did not increase when a 5 min “chase” followed the metabolic label (Supplementary Fig. 4d, e), suggesting that, at least over short time scales, there was no significant contribution of somatically synthesized RPs to the measured dendritic nascent protein signal. Taken together, these data indicate that RP mRNAs are locally translated in the neuropil and dendrites of hippocampal neurons.

### Dynamic association of nascent RPs with mature ribosomes

**Fig. 3:**
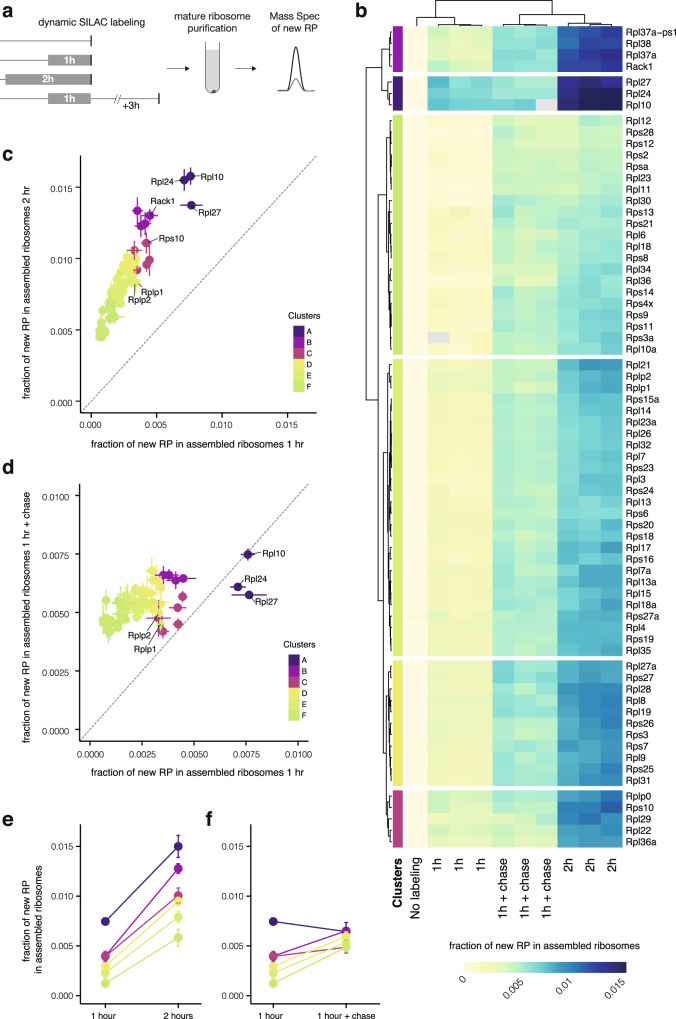
Some RPs rapidly incorporate into neuronal ribosomes. **a** Schematic of the experimental design for measuring new RPs in assembled ribosomes from cultured neurons. Normal medium was replaced by medium containing heavy amino acids (dynamic SILAC) as indicated (gray boxes). Mature ribosomes were purified by sucrose cushion. New ribosomal proteins were quantified by mass spectrometry, measuring the heavy and light peak of each peptide. **b** Heatmap showing for each RP the fraction of new proteins (*H*/(*H* + *L*)) incorporated into assembled ribosomes. Pseudocells (median of peptides obtained per individual protein) are ordered according to unsupervised clustering, both for columns (biological replicate of each condition) and rows (individual ribosomal protein). Experimental conditions of the labeling are indicated at the bottom. **c**, **d** Scatterplots showing the fraction of new RPs (*H*/(*H* + *L*)) in assembled ribosomes after the different labeling conditions, as indicated by *x*- and *y*-axes. Points represent average ± standard deviation of three biological replicates. Proteins are colored according to clusters identified in Fig. 3b. Some RPs of interest are indicated by name. Dashed line represents *x* = *y*. Source data are provided as a Source Data file. **e**, **f** Average ± standard deviation of the fraction of new proteins in assembled ribosomes of RPs of the same cluster, as identified in Fig. 3b. The different labeling conditions are indicated on the *x*-axis. Cluster A, *n* = 3 RPs. Cluster B, *n* = 4 RPs. Cluster C, *n* = 5 RPs. Cluster D, *n* = 11 RPs. Cluster E, *n* = 26 RPs. Cluster F, *n* = 21 RPs.

The dendritic synthesis of RPs, remote from the perinuclear region, prompted us to investigate the dynamics of RP association with mature neuronal ribosomes. We followed the incorporation kinetics of individual RPs into assembled ribosomes, asking whether all individual RPs are incorporated at an equal rate or whether there is a sub-population that is incorporated with different kinetics. To do so, we metabolically labeled newly synthesized proteins by incubating neurons for 1 or 2 h with heavy amino acids (dynamic SILAC,^37–40^). We then purified translating ribosomes using a sucrose cushion (optimized from^41^) and used mass spectrometry to quantify the fraction of new RPs present in assembled ribosomes (Fig. 3a). A sample without heavy amino acids served as negative control. We verified the translating status of the ribosomes by confirming the sensitivity of our preparation to conditions that disassemble monosomes and polysomes into free small and large subunits (the absence of magnesium or in the presence of the magnesium chelating agent EDTA, Supplementary Fig. 6a, b). Note that as the average half-life of a brain RP is ~8 days^42–44^, only ~0.4% of each protein is expected to be synthesized after 1 h of labeling (Supplementary Fig. 6c). Using a targeted mass spectrometry method (Parallel Reaction Monitoring^45^) to maximize our sensitivity, we reliably quantified 70 new RPs after 1 or 2 h of labeling (Fig. 3b and Supplementary Fig. 6d). For all RPs, we observed an increase in nascent RP incorporation into assembled ribosomes with increased labeling time (1 vs. 2 h) and the labeling level of individual RPs was significantly correlated between the two timepoints (*r*^2^ = 0.84, *p* < 0.0001; Fig. 3c). Importantly, the omission of the heavy amino acids led to a complete loss of the heavy peptide peak at the expected position (Supplementary Fig. 6d). The heavy signal was also drastically reduced when the cushion was performed in the presence of EDTA, further validating the specificity of our labeling for new translating ribosomes (Supplementary Fig. 6e–g). In addition, there was no significant difference in the ribosome association between nascent RPs that comprise large and small ribosome subunits (Supplementary Fig. 7a–c). Interestingly, however, individual nascent RPs exhibited distinct kinetics of accumulation in mature ribosomes, with the labeling fraction varying by >3.5-fold (Fig. 3b–e). To identify RPs that share similar kinetics we performed an unsupervised hierarchical clustering and detected 6 RP groups (Fig. 3b). Three groups (comprising a total of 12 proteins) exhibited a higher association level with assembled ribosomes (“rapidly incorporating”, clusters A-B-C in Fig. 3b–f) than the other three groups of RPs (comprising 58 proteins) (clusters D-E-F in Fig. 3b–f). This higher association rate of the rapidly incorporating group was detected following both 1 and 2 h of labeling (Fig. 3b–d). Within this group, we noted the presence of several RPs known to associate late during ribosome biogenesis, like RACK1 and RPL10^6,46^ validating the sensitivity of our measurements to the established temporal dynamics of ribosome assembly (Supplementary Fig. 7d, e).

To measure the potential time-lag between an individual RP’s synthesis and its association with mature ribosomes we performed a pulse-chase experiment. We labeled nascent RPs for 1 h and imposed a 3 h (label-free) chase before the purification of assembled ribosomes (Fig. 3a). Consistent with a time delay between synthesis and incorporation, the addition of the chase period led to a selective increase in mature ribosome association for the nascent RPs that showed slower incorporation kinetics (clusters D-E-F) in the previous 1 and 2 h labeling experiments (Fig. 3b–f). Interestingly, the remaining clusters (A-C) exhibited lower levels of incorporation after the chase. RPL27, RPL10 and RPL24 (cluster A), which showed the highest incorporation after 1 h labeling (no-chase, Fig. 3d), actually decreased their association with mature ribosomes when the chase was imposed (Fig. 3f). This indicates that either they rapidly and persistently associate with mature ribosomes or that they are replaced (exchanged) by nascent (but un-labeled) proteins synthesized during the chase. Supporting the idea of RP exchange, we noted the presence of RPLP1 and 2 among the proteins with the lowest fold-change after the chase; these proteins are the only two known RPs to transiently associate and dissociate from mature ribosomes^47,48^. Taken together, these data reveal that the binding kinetics of individual RPs to neuronal ribosomes are not homogeneous, and that a subset of RPs can rapidly and dynamically incorporate into neuronal ribosomes. Importantly, we note that the 12/12 mRNAs of the rapidly incorporating RPs were detected in the neuropil RNA-seq dataset (Fig. 1c), and 11/12 were detected in the ribosome footprint dataset (Fig. 2a and Supplementary 3). Additionally, we visualized the dendritic localization of both the mRNA (Fig. 1e and Supplementary Fig. 2) and the nascent protein (Fig. 2b and Supplementary Fig. 5), for all tested members of this group.

### Canonical biogenesis-independent incorporation of nascent RPs

**Fig. 4:**
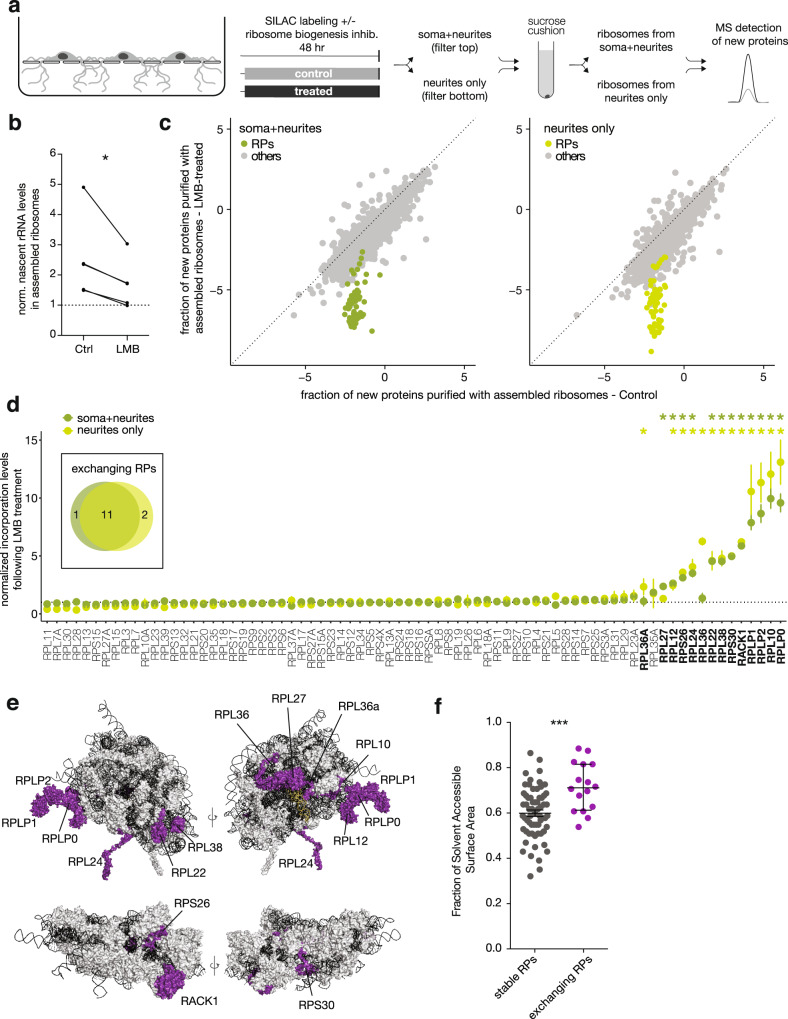
Some nascent RPs can incorporate into neuronal ribosomes independent of the canonical ribosome biogenesis pathway. **a** Schematic of the experimental design. Neurons were cultured on porous membranes where neurites can grow through the pores and be separately harvested. Normal medium was replaced by medium containing heavy amino acids for 2 days (filled boxes) in the presence (treated) or absence (control) of Leptomycin B (LMB) to prevent the export of nascent ribosomes from the nucleus and thus inhibit ribosome biogenesis. Mature ribosomes were purified from either compartment (top: cell bodies + neurites; bottom: neurites only) and new RPs were quantified by mass spectrometry as before. **b** Quantification by RT-PCR of new 18S rRNA in assembled ribosomes, relative to a non-labeled control (*y* = 1, dashed line). Two days of Leptomycin B (LMB) treatment significantly reduced the nascent rRNA levels in purified ribosomes (five paired biological replicates, paired *t*-test, two-tailed *p = 0.037). **c** Scatterplots showing fraction of new proteins (log_2_ of the *H*/*L* ratio) purified with assembled ribosomes from the cell bodies+neurites (left panel) or neurite-enriched (right panel) compartments of control or LMB-treated neurons. Ribosomal proteins are colored in green, all other proteins are indicated in gray. Dashed lines represent *x* = *y*. **d** Fold-change of the fraction of new RPs (*H*/(*H* + *L*)) detected in assembled ribosomes between control and LMB-treated samples, normalized to the median value obtained for each subunit (40S or 60S) (see methods). Ribosomes were purified from the cell bodies+neurites (dark green) or neurite-enriched (light green) compartments. Each point represents the mean ± standard deviation of four biological replicates. Exchanging RPs were identified as significant outliers by ROUT Method (*FDR ≤ 0.2%). See methods for information on statistical testing. Insert: Venn diagram of exchanging RPs in the two compartments. Source data are provided as a Source Data file. **e** Structure of human ribosome showing two views each for the large (top) and small (bottom) subunit (PDB: 4v6x). rRNAs in black, tRNA in yellow, stable RPs in gray, exchanging RPs in purple. Names are indicated for the exchanging RPs, as identified in Fig. 3d. **f** Fraction of solvent-accessible surface area for stable (*n* = 64 chains) and exchanging RPs (*n* = 16 chains), calculated for individual proteins within the structure of individual subunits of the human ribosome (PDB: 4v6x). Median ±  interquartile range. Mann–Whitney test, two-tailed ***p = 0.0007.

To test whether the above observed dynamic incorporation might represent the exchange of some nascent RPs on mature ribosomes, we determined the sensitivity of the rapid RP association to inhibition of ribosome biogenesis. We used leptomycin B (LMB)^49^ to block CMR1-mediated nuclear transport (Supplementary Fig. 8a, b), resulting in a sequestration of RPs (Supplementary Fig. 8c, d) and rRNA (Supplementary Fig. 8e, f) in the nucleus. To test whether LMB treatment effectively inhibited the export of nascent ribosomes, we incubated neurons with a uridine-analog to metabolically label nascent RNA and measured the levels of new rRNA (18S) (after sucrose cushion) to quantify new assembled ribosomes. Indeed, the level of nascent rRNA in mature ribosomes was significantly reduced in the present of LMB (Fig. 4b).

With LMB as a validated tool to interfere with canonical ribosome biogenesis, we addressed the spatial location of the RP-ribosome dynamics. To do so, we used compartmentalized chambers^50,51^, which separate and enrich a population of dendrites/axons that can be compared to a mixed cell-body + neurites population that resides above a porous membrane (Fig. 4a and Supplementary Fig. 7g–j). We labeled newly synthesized proteins by SILAC for 48 h in the presence or absence of LMB and then purified translating ribosomes from both compartments and measured the fraction of new proteins by mass spectrometry (Fig. 4a). LMB treatment did not affect global protein levels in the whole-cell lysate (Supplementary Fig. 9a–c), but resulted in a small decrease in total protein synthesis (Supplementary Fig. 9b–d), reproduced also by polysome profiling (Supplementary Fig. 9e, f). Consistent with the average half-life for neuronal ribosomes of ~8 days^42–44^, 48 h of LMB treatment resulted only in a small decrease of the total assembled ribosomes level (Supplementary Fig. 10a–c), but in a strong and specific reduction of new ribosomes (Fig. 4 and Supplementary Fig. 10b–d). In particular, we noted that the large subunit was more affected than the small subunit (Supplementary Fig. 10e). Taken together these data confirmed the successful inhibition of ribosome biogenesis via LMB treatment, prompting us to examine the association of nascent RPs with mature translating ribosomes. We found that while the association of most new RPs was equally reduced by LMB treatment, there was a subset of (~12) nascent RPs (putative exchangers) that again exhibited a significantly elevated association with translating ribosomes obtained from mixed somata + neurites (dark green data in Fig. 4d). Notably, we did not observe any difference in mRNA half-life between the putative exchangers and the other RPs (ref. ^31^, Supplementary Fig. 11a), indicating that a potentially differential depletion of RP mRNAs following nuclear export inhibition cannot explain the observed distinctive effect of LMB treatment on RP incorporation. As expected, within this group we again identified RPLP1 and 2, which are known to dynamically associate with the ribosome in the cytoplasm^52^ and RACK1, which has been recently shown to transiently interact with the ribosome in vitro^53^. The remainder of the exchanging RPs included RPLP0, RPL10, RPL22, RPL24, RPL38, RPS26, the same RPs that exhibited rapid incorporation in our previous experiment (Supplementary Fig. 11b).

When we examined the neurite fraction, we found that a largely overlapping group of RPs also exhibited evidence for biogenesis-independent exchange (light green data in Fig. 4d). Interestingly, 3 RPs were significantly different in either the mixed (somata + neurites; RPL27) or neurite-enriched compartment (RPL36 and RPL36a) (Fig. 4d), suggesting differential exchange of RPs could depend on specific subcellular environments. Additionally, among the common exchangers, several RPs showed a higher incorporation in the neurite compartment, where the contribution of dendrites and axons is not diluted by the cell bodies. Although we cannot unambiguously assert that the incorporation occurs locally, these observations are consistent with the idea that neurite-synthesized RPs can be locally incorporated into pre-existing ribosomes, and that at least the recruitment of these proteins is location-specific.

The above described exchange of RPs could endow neurons (and other cells) with the ability to repair or remodel ribosomes in situ (e.g., ref. ^54^) while avoiding the long time delays and the high energetic costs of degrading and producing a whole new ribosome^55,56^. In this regard, we noted a significant negative correlation between the level of incorporation of an RP and its half-life in both cultured neurons and intact brain, with exchanging RPs often exhibiting the shortest half-lives (Supplementary Fig. 11c, d,^43,44^). As ribosomal proteins are believed to be degraded when not assembled into ribosomes^57,58^, the shorter half-life of exchanging RPs might be due to their faster turn-over in the unbound state. In addition, the exchanging RPs identified here were present at sub-stoichiometric levels in individual ribosomal subunits, but not in monosomes and polysomes, quantified in heterologous cells^59^ (Supplementary Fig. 11e), suggesting that RPs might undergo exchange before the ribosomal subunits engage with the mRNA. Furthermore, we evaluated the position of exchanging RPs in the small and large subunits and noted that exchanging RPs were more surface exposed (Fig. 4e, f). These data indicate that most of the exchanging RPs detected here differ from other RPs in their half-lives, occupancy levels, and position in the mature ribosome. Finally, among the putative exchangers, 7 RPs (RACK1, RPS30, RPS26, RPLP0, RPL10, RPL24, and RPL36A) were reported in other systems to associate with immature ribosomes during the cytosolic phase of canonical biogenesis, and 5 (RPL38, RPL22, RPL12, RPL27, and RPL36) during the nuclear phase^6^. Our results suggest that this latter group can also associate with ribosomes in cytoplasmic compartments. Taken together, these data indicate that neurons can exploit the spatial and functional domains of ribosome assembly to dynamically incorporate RPs.

### Physiological modulation of RP incorporation

**Fig. 5:**
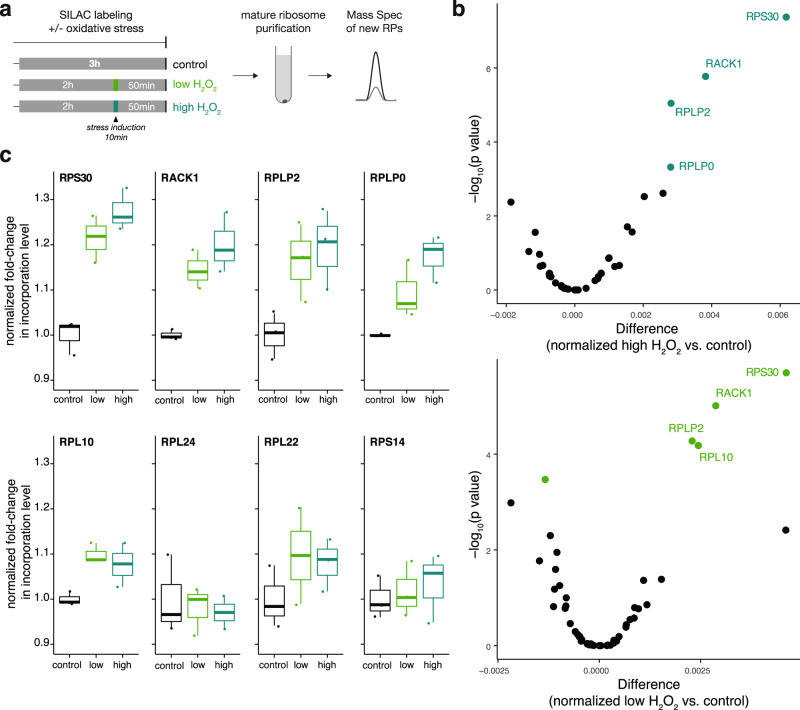
The association of a subset of exchanging RPs is relatively enhanced after oxidative stress. **a** Schematic of the experimental design for measuring new RPs in assembled ribosomes after oxidative stress. Normal medium was replaced by medium containing heavy amino acids (SILAC) for a total of 3 h. To induce stress, H_2_O_2_ was added for 10 min to a final concentration of 0.1 mM (light green) or 1 mM (dark green) to the heavy medium, 2 h after the beginning of labeling. Mature ribosomes were purified and new RPs were quantified by mass spectrometry as before. **b** Volcano plots of significantly regulated ribosomal proteins (green, FDR < 0.01) in assembled ribosomes, comparing control and H_2_O_2_-treated neurons (see methods for more detailed information on normalization and statistical testing). Source data are provided as a Source Data file. **c** Normalized fold-change in incorporation levels (see methods) for representative ribosomal proteins, which are significantly regulated (RPS30, RACK1, RPLP2, RPLP0, and RPL10) or not (RPL24, RPL22, and RPS14) with H_2_O_2_ treatment. Three biological replicates. Center of the box plots represents the median, hinges include first and third quartiles, and whiskers extend up to the smallest/largest value included in 1.5-fold the interquartile range (IQR). Source data are provided as a Source Data file.

The observation that RP exchange differs in subcellular compartments led us to investigate whether exchange is also regulated by different cellular states. In particular, we examined RP incorporation after a short induction of oxidative stress, via H_2_O_2_ incubation. As ribosomal proteins can be highly oxidized^60^ and oxidative stress changes neuronal function^61^, we reasoned that oxidative stress could enhance the need for ribosome repair. Additionally, oxidative stress rapidly leads to a translation reprogramming, where the synthesis of most proteins is transiently inhibited concomitant with the enhanced translation of specific stress-response transcripts^62–64^. To rapidly induce oxidative stress, we incubated neurons with H_2_O_2_ for 10 min, during a 3 h SILAC incubation to label newly synthesized proteins (Fig. 5a). Consistent with the general inhibition of translation, we found that the overall fraction of new RPs in assembled ribosomes was reduced during stress (Supplementary Fig. 12a). However, the association of a small subset (~4) of exchanging RPs was relatively enhanced after oxidative stress (Fig. 5b, c and Supplementary Fig. 12b, c) while the incorporation of the other exchangers (e.g., RPL24 and RPL22, Fig. 5c) was not differentially regulated. Altogether our data indicate that the incorporation probability of different RPs can change according to subcellular environments and physiological conditions.

## Discussion

Using a combination of methods, we describe here the localization and translation of ribosomal protein mRNAs in the dendrites and/or axons of neurons. Using dynamic SILAC and a targeted mass spec approach, we measured the incorporation rate of individual nascent RPs into mature ribosomes and identified a subset of ~12 RPs that exhibited an atypical rapid association with the ribosome. The rapid incorporation of these 12 RPs persisted when ribosome biogenesis was inhibited, providing strong evidence for the exchange of these of RPs on pre-assembled mature ribosomes. A recent study in *Xenopus* retinal ganglion cells also identified a subset of nascent RPs that associate with ribosomes within axons and the local synthesis of at least one RP is required for axon branching^8^ (Supplementary Fig. 11f). Interestingly, although we (and others) have observed a large (~50–70) population of RP mRNAs in neuronal processes, under the conditions explored here we detected the dynamic exchange of 12 individual RPs. We note that infrequent incorporation events of new RPs with slower kinetics might be undetectable by our method, which involves extremely brief metabolic labeling and labor-intensive curation of individual RP nascent peptides. Additionally, under different physiological conditions (e.g., cell stress or plasticity), some latent localized RP mRNAs could be translated and associate with mature ribosomes. In this case, the large number of distally localized RP mRNAs represents a huge potential for differential RPs incorporation.

Our discovery of dynamic non-canonical incorporation of RPs into mature ribosomes suggests new regulatory scenarios for local translational control. In neurons the capacity for remote remodeling or repair of ribosomes could be particularly advantageous considering the enormous cell volume, most of which arises from dendrites and axons. In addition, as ribosomes are the most heavily oxidized class of proteins^60^ and dendrites are particularly sensitive to oxidative insults^65^, ribosomes in neuronal processes may be prone to higher proteotoxic damage, creating demand for the local repair of ribosomes.

The dynamic incorporation of RPs could also alter ribosome composition resulting in potentially “specialized” ribosomes. The rather long life of the ribosome as a macromolecular protein-RNA machine as well as its nucleo-centric biogenesis represent challenges for rapid remodeling. In other systems, ribosomes with altered stoichiometries of RACK1, RPL38, or RPS26 preferentially translate different subsets of mRNAs^66–68^. Such specialization could be rapidly achieved through the dynamic exchange of these proteins. This property could be particularly exploited by ribosomes in dendrites and axons, which are optimally positioned to respond to synaptic signals that could, in principle, remodel local ribosomes as well as the local translatome.

## Methods

### Preparation of primary cultured neurons

Dissociated rat hippocampal or cortical neuron cultures were prepared and maintained as described previously^69^. Briefly, we dissected hippocampi or cortices from postnatal day 0 to 2 rat pups of either sex (Sprague–Dawley strain; Charles River Laboratories) and dissociated the samples with papain (Sigma). For imaging experiments, hippocampal neurons were plated at a density of 30 × 10^3^ cells/cm^2^ on poly-Dlysine–coated glass-bottom petri dishes (MatTek). For biochemical experiments, cortical neurons were plated at a density of 4 × 10^6^ cells/cm^2^ on poly-Dlysine-coated 10 cm dishes, at a density of 1 × 10^6^ cells/cm^2^ on poly-Dlysine-coated 6 cm dishes or at a density of 9 × 10^6^ cells/cm^2^ on poly-Dlysine-coated 75 mm inserts (3.0 µm pore size, Corning 3420). One day after plating on the inserts, cells were incubated with 5 µM AraC (Sigma C1768) for 2 days, then the AraC was removed by changing the media. Neurons were maintained in a humidified atmosphere at 37 °C and 5% CO_2_ in growth medium [Neurobasal-A supplemented with B27 and GlutaMAX-I (Life Technologies)] for 14–16 DIV for biochemical experiments or for 25–27 DIV for imaging experiments. Housing and sacrificing procedures complied with German national and international animal care policies and the guidelines issued by the Max Planck Society (DIRECTIVE 2010/63/EU; German animal welfare law; FELASA guidelines) and were approved by local authorities (Regierungspräsidium Darmstadt).

### Pharmacological treatments

To inhibit ribosome biogenesis, cells were treated with 20 µg/µL Leptomycin B (Merck, 431050) for 2 days, unless otherwise specified. To inhibit proteasome degradation, cells were treated with 10 µM MG132 (Sigma, M8699) for at least 2 h. To induce oxidative stress, cells were incubated with 1 mM or 0.1 mM H_2_O_2_ (AlfaAesar L13235) for 10 min.

### Metabolic labeling of newly synthesized proteins by dynamic SILAC

Heavy medium was prepared by adding 84 mg/L of Arg10 (ThermoFisher 88434) and 146 mg/L of Lys8 (ThermoFisher 88432) into a Arg- and Lys-free Neurobasal-A medium (ThermoFisher, customized). To condition the medium, extra plates from each prep were incubated with the same medium starting from DIV 0. On the day of the dynamic SILAC experiment (DIV 13–16), the conditioned heavy medium was collected from the extra plates. The original “light” medium was then replaced by the conditioned heavy medium for the indicated amount of time (1 h, 2 h, or 2 days). To reduce the likelihood of purifying polypeptide chains still emerging from the ribosome, a 5 min wash with light medium was used to allow termination of translation after the 1 h or 2 h labeling.

### Ribosome purification by sucrose cushion

Cells were washed three times and scraped in ice-cold DPBS (ThermoFisher, 14040-091) supplemented with 100 µg/mL of CHX (Sigma, C7698). An aliquot was saved to prepare a whole-cell lysate. Cells were pelleted by 5 min centrifugation at 500 × *g*. For ribosome purification, cells were lysed in 400 µL of ribosome lysis buffer (20 mM Tris pH 7.4, 150 mM NaCl, 5 mM MgCl_2_, 24 U/mL TurboDNase, 100 µg/mL cycloheximide, 1% Triton-X-100, 1 mM DTT, RNasin(R) Plus RNase inhibitor 200 U/mL and 1x cOmplete EDTA-free protease inhibitor). For the compartmentalized chambers, first the top compartment (cell bodies + neurites) was scraped in DPBS + CHX and then the bottom (neurites only) was scraped directly in 200 µL of ribosome lysis buffer. To guarantee sufficient yield from the neurite compartment, each biological replicate consisted of the content of two inserts pooled together. Lysates were pipetted up and down until homogenization was clear with a 0.4 × 20mm syringe needle (HSW FINE-JECT) on ice. Samples were then centrifuged at 10,000 × *g* for 10 min at 4 °C. Supernatants were loaded on 1 mL sucrose solution (34% sucrose, 20 mM Tris pH 7.4, 150 mM NaCl, 5 mM MgCl_2_, 1 mM DTT, 100 µg/mL cycloheximide) in a thickwall polycarbonate tube (Beckman, 349622) and centrifuged for 30 min at 4 °C at 55,000 rpm (367,600 × *g*) with a SW55Ti rotor (acceleration 0, deceleration 7). Ribosome pellet was resuspended in 20 µL of 10 mM HEPES, 120 mM NaCl, 3 mM KCl, 10 mM D-Glucose, 2 mM MgSO4 and 2 mM CaCl_2_, and submitted to Mass Spectrometry or Western Blot Analysis. For the experiment shown in Supplementary Fig. 6a, b, e–g, ribosome lysis buffers and cushion solutions were modified to either contain 0 mM MgCl_2_ or 5 mM MgCl_2_ + 15 mM EDTA. After the ribosome pellet resuspension, the same volumes across samples were used for downstream analyses (western blot or mass spectrometry).

### Ribosome purification by polysome profiling

Cells were processed as for sucrose cushion, except the ribosome lysis buffer was supplemented with 8% glycerol and the supernatant were loaded on a 10–50% sucrose gradient. For the gradients, all solutions were prepared in gradient buffer (20 mM Tris pH 7.5, 8% glycerol, 150 mM NaCl, 5 mM MgCl_2_, 100 µg/mL cycloheximide, 1 mM DTT). Gradients were prepared by sequentially adding solutions with different sucrose concentrations (in order from first added to last, 8 mL of 55%, 0.5 mL of 50%, 0.5 mL of 40%, 0.5 mL of 30%, 0.5 mL of 20%, 0.5 mL of 10%) into the same Thinwall polypropylene tube (Beckman, 331372). Tubes were placed at −80 °C to freeze the content before adding the next sucrose solution, and finally stored at −80 °C. The day prior to experiments, gradients were left for equilibration at 4 °C overnight. Then 1 to 2 OD (measured with NanoDrop at 260 nm) of the lysates were loaded on top of the gradients and spun at 36,000 rpm (222,200 × *g*) at 4 °C for 2 h with a SW41-Ti rotor (Beckman). Gradients were then run at 850 µL/min in a density gradient fractionation system (Teledyne Isco), chased by 60% sucrose 10% glycerol in water. RNA absorbance at 254 nm was continuously measured using a UA-6 detector. The area under the curve corresponding to the monosome and polysomes was measured. To compare across different runs, the polysome fraction was calculated as area under polysomes over the sum of the areas under monosome and polysomes.

### Total-cell lysates

Cell were scraped as described above for sucrose cushion. Cell pellets were lysed in 200 µL of 8 M urea, 200 mM Tris/HCl [pH 8.4], 4% CHAPS, 1 M NaCl, cOmplete EDTA-free protease inhibitor (Roche, 11873580001), using a pestle. Lysates were sonicated at 4 °C for four rounds of 30 s each, and incubated for 10 min with 1 µL of Benzonase (Sigma E1014). After centrifugation for 5 min at 10,000 × *g*, the supernatant was submitted to mass spectrometry or western blot validation.

### FISH in hippocampal slices

In all, 3–5-week-old Sprague–Dawley SPF rats were housed on a 12/12-h light/dark cycle with food and water ad libitum until euthanasia. Animals were anesthetized by Isoflurane inhalation (Abbott, USA). The rat head was removed and immediately frozen in liquid nitrogen for 30 s. The brain was extracted and sliced into 500–600 µm slices in ice-cold oxygenated sucrose-ACSF using a vibratome (VT1200S, Leica, Germany). The slices were fixed in a fixation buffer (4% PFA, 4% sucrose in PBS) for 1 h at 4 °C and then 1 h at room temperature. After washing with PBS, slices were dehydrated with ice-cold 15% sucrose in PBS for 2–3 h at 4 °C and then ice-cold 30% sucrose in PBS overnight at 4 °C. Slices were blocked in O.C.T. (SAKURA Finetek USA Inc., USA) and sliced again at 30 µm thickness using a sliding microtome (Microm HM450, ThermoFisher), followed by thorough washing with PBS and fixation for 20 min at room temperature using a fixation buffer (4% paraformaldehyde, 5.4% glucose, 0.01 M sodium metaperiodate in lysine-phosphate buffer). In situ hybridization was performed using the ViewRNA ISH Cell Assay Kit (ThermoFisher) according to the manufacturer’s instructions with some modifications. Briefly, slices were permeabilized at room temperature using the detergent solution for 20 min. After washing with PBS and 5 min incubation with the hybridization buffer, the respective probes (see Supplementary table 1 for details) were diluted 1:100 in the pre-warmed working hybridization buffer and added to the slices. After incubation at 40 °C overnight and washing with the wash buffer, 1:100 PreAmplifier Mix was diluted in pre-warmed working amplifier diluent and incubated with the slices for 1 h at 40 °C, followed by 1 h incubation with 1:100 Amplifier mix in pre-warmed working amplifier diluent and then 1 h with 1:100 Label probe mix in pre-warmed working label probe diluent. After washing, the slices were permeabilized with 0.5% Triton-X-100 in blocking buffer (4% goat serum in PBS) for 20 min and blocked in blocking buffer for 1 h. Immunostaining was then performed using antibodies against Map2 (see Supplementary table 2 for details) overnight at 4 °C, followed by secondary antibody donkey anti-gp Cy5 (1:500, 706-175-148, Dianova, Germany) and 1:1000 DAPI for 2 h at room temperature. Mounting of slices was performed using Aqua Poly/mount (18606, Polysciences, USA).

### FISH in hippocampal cultures

Cultured rat hippocampal neurons (DIV 21–28) were fixed for 20 min at room temperature using a fixation solution (4% paraformaldehyde, 5.4% glucose, 0.01 M sodium metaperiodate in lysine-phosphate buffer). In situ hybridization was performed using the ViewRNA ISH Cell Assay Kit (ThermoFisher) according to the manufacturer’s instructions with some modifications. Briefly, neurons were permeabilized at room temperature by treating with detergent solution for 5 min, followed by pepsin digestion (0.01 mg/mL of enzyme in 10 mM HCl) for 45 s. After washing with PBS, the respective probes (see Supplementary Table 1 for details) were diluted 1:100 in pre-warmed hybridization buffer and added to neurons. After incubation at 40 °C for 3 h, neurons were washed with wash buffer and stored in storage buffer overnight at 4 °C. After several washes, neurons were incubated for 30 min at 40 °C with PreAmplifier Mix (diluted 1:25 in pre-warmed working amplifier diluent), followed by 30 min incubation with 1:25 Amplifier mix in pre-warmed working amplifier diluent and then 30 min with 1:25 Label probe mix in pre-warmed working label probe diluent. After washes, neurons were immunostained with antibodies against Map2 (see Supplementary Table 2 for details) overnight at 4 °C, followed by secondary antibody donkey anti-gp Cy5 (1:500, 706-175-148, Dianova, Germany) for 1 h at room temperature and DAPI for 5 min.

### Puro-PLA

Detection of newly synthesized proteins by puromycin labeling and proximity ligation was performed as previously described^36^. Neurons were metabolically labeled for 5 min with 1 µM puromycin (Sigma, P8833). For the chase experiment (shown in Supplementary Fig. 3d, e), after the 5 min labeling cells were washed three times and incubated for 5 more minutes with the original medium. All samples were washed twice with DPBS (ThermoFisher, 14040-091) prior to fixation (20 min in 4% PFA in 4% sucrose in PBS). Cells were permeabilized (15 min in blocking buffer + 0.5% Triton-X-100) and blocked (>30 min in blocking buffer, PBS + 4% goat serum). Neurons were incubated overnight at 4 °C in PBS + 4% goat serum containing primary antibodies against puromycin, the protein-of-interest and MAP2 to label dendrites (see Supplementary Table 2 for details). After washing, proximity ligation assay (PLA) was performed using the Duolink In Situ PLA kit (Sigma). In particular, PLA probes anti-rabbit PLUS (DUO92002) and anti-mouse MINUS (DUO92004) and the Duolink Detection reagents Red (Sigma DUO92008) were used according to the manufacturer’s recommendations. Briefly, probes (1:10 dilution) and a secondary antibody for MAP2 were applied in PBS with 4% goat serum for 1 h at 37 °C, washed three times with wash buffer A (0.01 M Tris, 0.15 M NaCl, 0.05% Tween 20) and incubated for 30 min at 37 °C with the ligation reaction. Samples were then washed three times with wash buffer A and incubated at 37 °C for 100 min with the amplification reaction mixture. Amplification was stopped by three washes in wash buffer B (0.2 M Tris, 0.1 M NaCl, pH 7.5). Nuclei were stained with DAPI (1:1000 for 2 min) and cells were kept in wash buffer B at 4 °C until imaging.

### Image acquisition and analysis for Puro-PLA and FISH

Within a week after labeling, samples were imaged using a LSM780 confocal microscopy (Zeiss), software ZEN 2.3 SP1 FP3 (version 14.0.23.201) and a Plan-Apochromat 40x/1.4 Oil DIC M27 objective. Neurons were identified as MAP2-positive cells, and they were selected for imaging and quantification if the majority of their dendritic arbor could be unambiguously assigned to one cell body. z-stack was set to cover the entire volume of a neuron, with optical slice thickness set to optimal. Laser power and detector gain were adjusted to avoid saturated pixels. Imaging conditions were held constant within experiments. Maximum intensity projections of image z-stacks were used for image analysis. For visualization purposes (but not for analysis), the punctae size was dilated and brightness and contrast adjusted. Image analysis was performed in ImageJ/FIJI with an in-house script. For cell culture, the dendritic arbor and the cell body of individual neurons were manually traced using the MAP2 immunolabeling. For hippocampal slices, the somatic compartment was defined by a 5 µm dilation of the DAPI signal. After thresholding, the intensity and number of punctae were quantified and normalized over the annotated neuronal area.

### Immunofluorescence

Cells were fixed for 20 min in 4% PFA in 4% sucrose in PBS, permeabilized for 15 min in 0.5% Triton-X-100 + blocking buffer and blocked for at least 30 min in blocking buffer (PBS + 4% goat serum). Neurons were incubated for 2 h with primary antibodies and, after three washes, for 1 h with secondary antibodies, all in blocking buffer (see Supplementary table 2 for antibodies information). After two washes in PBS, cells were stained with DAPI (1:1000 for 2 min) and kept in PBS at 4 °C until imaging. For validation of the compartmentalized chambers, pieces of the filter were processed as described above, and mounted on a glass slide (ThermoFisher 10417002) with Aqua Poly/mount (Polysciences, 18606). Samples were imaged using a LSM780 confocal microscopy (Zeiss) using a Plan-Apochromat 40x/1.4 Oil DIC M27 or Plan-Apochromat 20x/0.8 M27 objectives. A z-stack was set to cover the entire volume of neurons, with optical slice thickness set to optimal. Laser power and detector gain were adjusted to avoid saturated pixels. Imaging conditions were held constant within experiments. Maximum intensity projections of z-stacks were used for image analysis. For visualization purposes (but not analyses) brightness and contrast were adjusted.

All image analyses were performed in ImageJ/FIJI with a fully automated script built in-house. In Supplementary Fig. 6a, b, the intensity of RanBP1 signal was quantified within two masks, containing the whole nucleus with or without the outer edge (representing the nuclear envelope). The fraction of RanBP1 within the nucleus was calculated as the signal in the inner mask, over the signal in the outer mask. In Supplementary Fig. 6e, f, the intensity of the Y10b signal was quantified within a mask based on the Nucleolin channel, which was co-stained to label nucleoli. In Supplementary Fig. 6g, h, the number of nuclei was quantified based on the DAPI channel.

Statistical testing was performed using GraphPad Prism (RRID:SCR_002798, version 9.1.0).

### Mass spectrometry

#### Sample preparation for MS analysis

Proteins were digested according to the ‘Filter-Aided Sample Preparation’ (FASP) protocol^70^ or using S-Traps according to an adapted version of the suspension trapping protocol described by the manufacturer (ProtiFi, Huntington, NY). Peptides were desalted using C18 StageTips^71^, dried by vacuum centrifugation and stored at −20 °C until Liquid chromatography–mass spectrometry (LC-MS) analysis.

#### LC-MS/MS analysis

The peptide samples were reconstituted in 5% acetonitrile (ACN) and 0.1% formic acid (FA) supplemented with an iRT peptide standard (1:10 dilution; Ref.: Ki-3002-2; Biognosys). Peptides were separated by nano-HPLC (U3000 RSLCnano, Dionex). The samples were loaded and washed with loading buffer (2% ACN, 0.05% trifluoroacetic acid (TFA) in water; 6 min; 6 µL/min) on a PepMap100 loading column (C18, *L* = 20 mm, 3 µm particle size, Thermo Scientific) and subsequently separated on a PepMap RSLC analytical column (C18, *L* = 50 cm, <2 µm particle size, Thermo Scientific) by a gradient of phase A (0.1% FA in water) and phase B (80% ACN, 0.1% FA in water). The gradient was ramped from 4% B to 48% B in 90 min at a flow rate of 300 nL/min. All solvents were purchased from Fluka in LC-MS grade. Eluting peptides were ionized online using a Nanospray Flex ion source (Thermo Scientific) and analyzed in a Q-Exactive Plus mass spectrometer (Thermo Scientific) (see Supplementary Data 2 for method details). In brief, for DDA mode, precursor ion spectra were acquired over the mass range 350–1400 *m*/*z* (mass resolution (*R*) = 70 k, AGC target 3 × 10^6^, maximum injection time (IT) = 60 ms). The top-10 precursor ions were selected for fragmentation (HCD; normalized collision energy = 30) and analyzed in MS2 mode (*R* = 17.5 k, isolation window = 1.7 Da, AGC target = 2 × 10^4^, maximum IT = 50 ms). In a parallel reaction monitoring (PRM) approach^45^, MS2 scans were acquired (*R* = 17.5 k, isolation window = 1.7 Da, AGC target = 1 × 10^5^, maximum IT = 64 ms) according to the scheduled inclusion lists.

#### MS-data processing

For protein identification and relative quantification of the DDA data, MS raw data were analyzed with MaxQuant (version 1.6.2.3 and 1.6.0.1; RRID:SCR_015753)^72,73^ using customized Andromeda parameters (see Supplementary Data 2 for LC + MS parameters and Supplementary Data 3 for MaxQuant settings). For all searches, spectra were matched to a Rattus norvegicus database (reviewed and unreviewed; downloaded from uniprot.org (RRID:SCR_004426)) considering tryptic peptides with up to 2 missed cleavages and to contaminant and decoy databases. Precursor mass tolerance was set to 4.5 ppm and fragment ion tolerance to 20 ppm. Carbamidomethylation of cysteine residues was set as fixed modification and protein-N-terminal acetylation, as well as methionine oxidation were set as variable modifications. A false discovery rate (FDR) of 1% was applied at the peptide-spectrum-match (PSM) and protein level. Only proteins identified by at least one unique peptide were retained for downstream analysis. For relative protein quantification, the data was searched with a multiplicity of 2 (light (Lys0, Arg0) and heavy (Lys8, Arg10)) and the LFQ values were computed without normalization.

For the targeted analysis of ribosomal proteins by PRM, raw data was analyzed in Skyline (version 20.1.0.155; RRID: SCR_014080)^74^. To obtain information on target peptides, a series of DDA scout runs was measured. Targeted peptides were selected based on uniqueness, no missed cleavages, recurrent occurrence and signal intensity. Given a high degree of sequence similarity amongst RPs, some ribosomal proteins could not be represented by more than one unique peptide. Peptide identity was confirmed using a spectral library generated in Skyline using the results of a MaxQuant search (msms.txt) with a multiplicity of 1 (only light) of the DDA data. In Skyline, a scheduled method was generated using target detection windows of 3 min, which was split into three inclusion lists to analyze separate injections measured with PRM methods 1–3. For final data curation, PRM raw data were imported as multiple-injection replicates in Skyline and peak picking was confirmed manually in accordance to retention time, mass accuracy and library matches.

All MS data associated with this manuscript have been uploaded to the PRIDE repository and are available with the dataset identifier PXD026973 (RRID:SCR_003411)^75^.

#### Protein quantification and statistical analyses

For targeted analysis of nascent ribosomal proteins after 1 h, 2 h, or 3 h of SILAC labeling, heavy and light peptide signals were curated in Skyline, peak areas were exported and heavy peptide fractions (%*H* = *H*/(*H* + *L*)) were calculated in R. Protein heavy fractions were determined by combining the peptide-specific heavy fractions by their median value. Protein fold changes were calculated as median of the corresponding peptide fold changes. For unsupervised clustering, protein heavy fractions were hierarchically clustered using Euclidean distance. Visualization of the cluster data was done using the pheatmap R-package (RRID: SCR_016418, https://CRAN.R-project.org/package=pheatmap). For the EDTA experiment (in Supplementary Fig. 6e–g), total intensity (*H* + *L*) was calculated for each peptide in R. Peptide intensity values were aggregated by their median and protein values were merged among biological replicates by their mean. Fold changes (EDTA vs. Control) were first calculated for each peptide then combined at the protein level.

For analysis of nascent ribosomal proteins after 2 days of SILAC labeling with or without LMB treatment, peptide signals of ribosomal proteins were manually curated in Skyline to ensure accurate quantification, especially for low abundant heavy peptides. Subsequently heavy peptide fractions were calculated in R. Technical replicates were merged (mean) and only peptides with a heavy fraction that was 3× greater in the SILAC samples (both the LMB treated and untreated condition) compared to the “no labeling” samples were used for downstream analysis. LMB-treated versus control fold changes of the heavy fractions were calculated on the peptide level and protein fold changes were determined as the median of the corresponding peptide fold changes. To correct for different size effect of LMB-treatment on the two ribosome subunits, the fold-change of each ribosomal protein was normalized over the median change of the corresponding subunit. Exchanging ribosomal proteins were identified as significant outliers by ROUT Method (*Q* = 0.2%) in GraphPad Prism (RRID:SCR_002798, version 9.1.0).

To analyze the effect after 2 days of SILAC labeling with or without LMB treatment on all proteins in the total lysate- or cushion-samples, MaxQuant results of the protein groups (proteinGroups.txt) were further processed in R. Protein groups were filtered to remove contaminant or decoy database hits and proteins only identified by a modified peptide (“identified only by site”). Total intensity (*H* + *L*) and heavy over light ratios (*H*/*L*) were log2-transformed and values of the technical duplicates were merged by their means. Subsequent principal component and Pearson correlation analyses were conducted in R using its base functions. Differential regulation comparing LMB-treated and control samples was investigated using unpaired, two-sided *t*-tests. To correct for multiple testing, Benjamini–Hochberg correction was applied with an FDR cutoff < 0.01.

For the analysis on the oxidative stress (low or high H_2_O_2_), we corrected for the general decrease in protein synthesis by normalizing the heavy fraction of each peptide in treated samples over the average fold-change between the treated and control samples of all peptides. Subsequently, we used the linear mixed effect model implemented in MSqRob^76^ to calculate the statistical significance of the differential incorporation between H_2_O_2_-treated and control samples. The treatment was set as fixed effect of interest, the different peptides of the same protein as random effects, and protein name as grouping factor. Half of the interquantile range of the average difference between the normalized treated samples and the controls was used as minimal difference for a comparison to be accepted as significant. To correct for multiple testing, Benjamini–Hochberg correction was applied with an FDR cutoff <0.01. Protein heavy fractions were determined by combining the peptide-specific heavy fractions by their median value and are reported in the Source Data. To visualize the fold-change relative to control (in Fig. 5c), each protein heavy fraction was normalized over the median of the average observed values in the control samples.

To analyze the protein composition of ribosomes across different translational states, an available polysome proteome profiling dataset was downloaded (ref. ^59^, PRIDE: PXD009292). The abundance (SILAC ratio over internal standard) of ribosomal proteins in selected fractions (40S, 60S, 80S, and polysome) was extracted and the value of each ribosomal protein was normalized over the median abundance of all proteins of the corresponding subunit within each fraction (when considering proteins of the small subunit, the fraction corresponding to the 60S was excluded, and vice versa for the large subunit and the 40S fraction). For unsupervised clustering, the normalized levels of ribosomal proteins across fractions were hierarchically clustered using Euclidean distance, and clusters were visualized using the pheatmap R-package (RRID: SCR_016418, https://CRAN.R-project.org/package=pheatmap).

### Structural analysis

The surface accessible areas of ribosomal proteins were calculated using the PDBePISA web service of the EBI (PDBe PISA v1.52 [20/10/2014], https://www.ebi.ac.uk/pdbe/pisa/pistart.html, Krissinel et Henrick, 2007) using the structure of small and large subunits of the human ribosome (PDB entry: 4V6X, https://www.rcsb.org/sequence/4V6X^5^). In brief, PDB data were imported using the biological assembly CIF file, and total surface and interface areas were calculated for each chain using standard parameters in PISA. The solvent-accessible surface areas (calculated from the total surface area and all interface areas, including RPs and rRNAs) values were extracted and summed up for every chain in the dataset. Statistical testing was performed using GraphPad Prism (RRID:SCR_002798, version 9.1.0).

### Detection of newly synthesized rRNA in assembled ribosomes

New RNA was labeled by incubating cells for 3 h with 5 mM EU (5-Ethynyl Uridine) (ThermoFisher, E10345). Assembled ribosomes were purified by sucrose cushion (see above) and the ribosome pellet was resuspended in 1 mL of TRIzol (ThermoFisher, 15596018). New RNA was purified by Click-iT Nascent RNA Capture Kit (ThermoFisher, C10365), according to the manufacturer’s recommendations. Briefly, 500 ng of RNA were clicked with 0.5 mM Biotin-Azide and EU-labeled RNA was purified by Dnyabeads MyOne Streaptavidin T1 beads. After washes, 1 µL of pre-diluted 1:200 ERCC RNA spike-in control mixes (ThermoFisher, 4456740) was added to all samples and reverse transcription was performed on the beads. The cDNA was then quantified by qPCR using TaqMan assay for the 18S rRNA (Thermofisher, Mm04277571_s1) and the ERCC-130 (5′-/5HEX/CGGAACAGG/ZEN/GCTGACGCCGC/3IANkFQ/-3′). To correct for differences in reverse transcription efficiency, each sample was internally normalized over ERCC-130 values. Finally, each experiment was normalized to a non-EU-labeled control.

### Live-cell imaging of RPL10a-PA-RFP

Cultured neurons were transfected at DIV 11–14, using the Magnetofectamine O2tm system, to express RPL10a tagged with a photoactivatable RFP (p323-L10A-PATagRFP, addgene plasmid #74172) and GFP as cell fill (pAcGFP1-C1, Clontech 632470). One or 2 days after transfection, neurons were imaged in supplemented E4 buffer (10 mM HEPES, 120 mM NaCl, 3 mM KCl, 10 mM d-Glucose, 2 mM MgSO_4_ and 2 mM MgSO_4_, 1x B27, 1x GlutaMax, 1x MEM amino acids) with an inverted spinning disk confocal microscope (Zeiss 3i imaging systems; model CSU-X1). Images were acquired with Plan-Apochromat 63x/1.4 Oil DIC objective, at 488 nm (5 mW laser power and 50 ms exposure) and 561 nm (20 to 30 mW laser power and 100 ms exposure), using the Slidebook (version 5.5.5; RRID:SCR_014300) software. Transfected cells were identified as GFP-positive. z-stack (with 0.63 µm increments) was set to cover the whole-cell body, and time-lapse was set with 30 min intervals. Photoactivation of a circular region around the nucleolus (identified by the lack of GFP signal) was performed using the 445 nm laser at 100 mW (two repetitions of 5 ms, at the center of the z plane). The sum intensity projection was used for image analysis. After background noise removal, the intensity inside the whole nucleus was quantified at each time point, and normalized over the maximum intensity reached after photoactivation. Statistical testing was performed using GraphPad Prism (RRID:SCR_002798, version 9.1.0).

### Western blot

Samples (total-cell lysates or sucrose cushion) were prepared as described above. After addition of NuPAGE LDS Sample Buffer (ThermoFisher, NP007) and NuPAGe Sample Reducing Agent (ThermoFisher, NP004) to a final concentration of 1x, samples were loaded onto 4% to 12% Bis-Tris NuPAGE gels (ThermoFisher). Gels were transferred using the Trans-Blot Turbo Transfer Pack (Biorad, 1704157) on a polyvinylidene fluoride membrane (Immobilon-FL, IPFL00010 0.45 µm pore size). Membranes were stained using Revert 700 Total Protein Stain (LI-COR, 926-11015) for loading normalization. Immunoblotting was performed with primary antibodies as indicated (see Suuplementary Table 2 for antibodies information) and secondary antibodies IRDye 680 and 800 (1:5000, LI-COR 926-68071, 926-68020, 926-32210, 926-32211). Images were acquired using LI-COR Image Studio Lite (version 3.0.30, RRID:SCR_013715) and analyzed using ImageJ/FIJI. Statistical testing was performed using GraphPad Prism (RRID:SCR_002798, version 9.1.0).

### Reporting summary

Further information on research design is available in the Nature Research Reporting Summary linked to this article.

## Supplementary information

Supplementary Information

Peer Review File

Description of Additional Supplementary Files

Supplementary Data 1

Supplementary Data 2

Supplementary Data 3

Reporting Summary

## Data Availability

The data that support this study are provided in the main text or the supplementary materials, and are available from the corresponding author upon reasonable request. The mass spectrometry proteomics data have been deposited to the ProteomeXchange Consortium via the PRIDE^75^ partner repository with the dataset identifier PXD026973. Source data are provided with this paper.

## Source data

Source data

## Code availability

Analysis scripts are available upon request.
