# Supplementary material for: Neuronal ribosomes exhibit dynamic and context-dependent exchange of ribosomal proteins
Source: Nat Commun. 2021 Oct 21;12:6127. doi: 10.1038/s41467-021-26365-x (PMC8531293; doi:10.1038/s41467-021-26365-x)
Supplement: Supplementary file 1 — Supplementary Information [file 41467_2021_26365_MOESM1_ESM.pdf]

Supplementary Information for

Neuronal Ribosomes exhibit dynamic and context-dependent exchange of ribosomal proteins.

Claudia M. Fusco[1], Kristina Desch[1#], Aline R. Dörrbaum[1,2#], Mantian Wang[1,3], Anja Staab[1], Ivy C.W. Chan[1,4], Eleanor Vail[1], Veronica Villeri[1,5], Julian D. Langer[1,6], Erin M. Schuman[1*]

* to whom correspondence should be addressed: erin.schuman@brain.mpg.de

The PDF files includes:

- Supplementary Figures
- Supplementary Tables
- Supplementary References

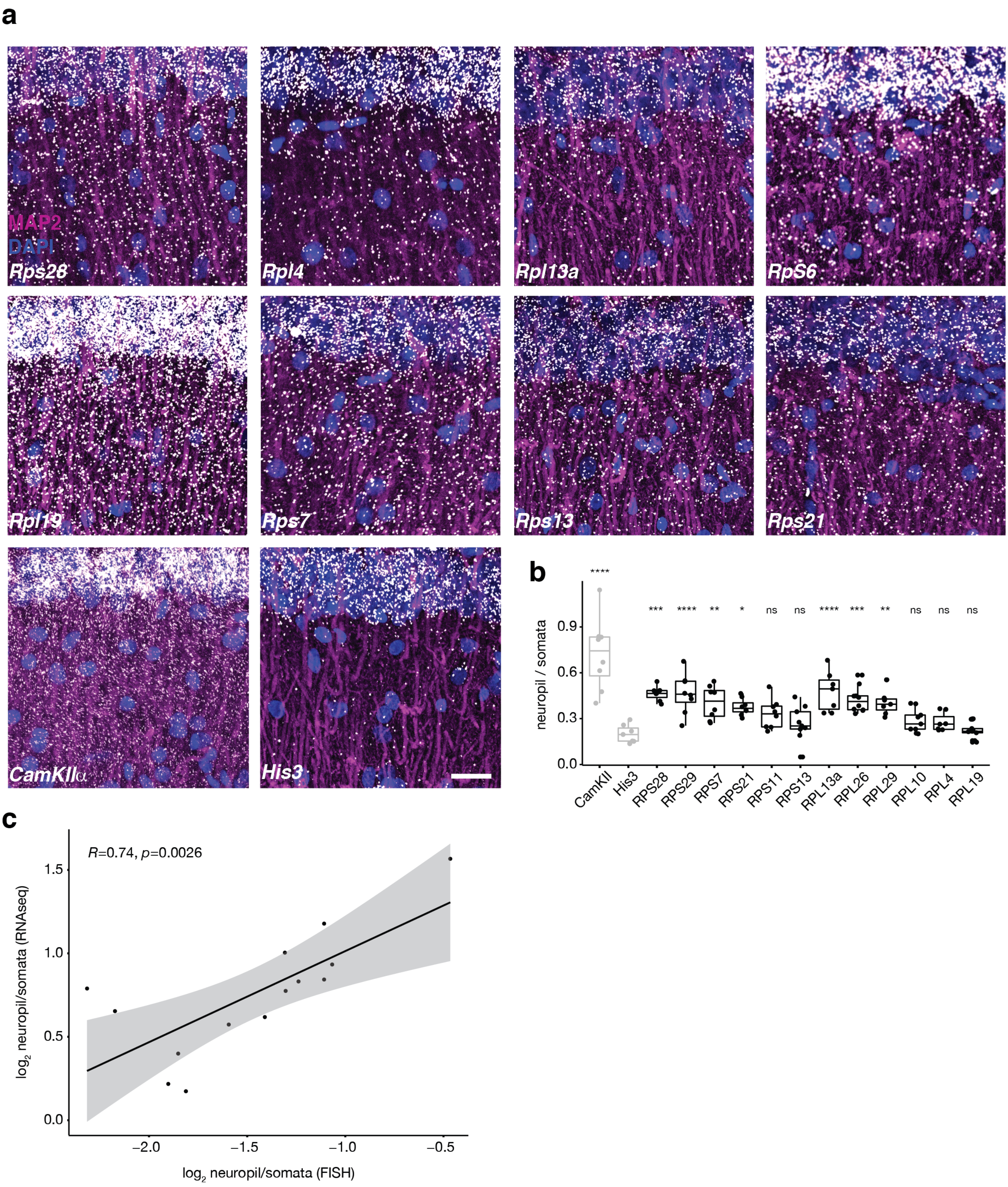

Supplementary Figure 1

Supplementary Figure 1: Detection of RP mRNAs in dendrites of rat hippocampal slices.

a, FISH detection of indicated RP mRNAs in dendrites (magenta MAP2, white FISH, blue DAPI) in hippocampal slices. Images are oriented with the somata layer at the top and the dendrites extending below. Scale bar = 50 μm. Representative images from n≥7 field of views, acquired over ≥2 biologically and technically independent experiments,

b, Quantification of the FISH signal between the neuropil and somata layers in hippocampal slices (Ordinary one-way ANOVA ($p<0.0001$) followed by a Dunnett's multiple comparison test using *His3* as control group, * p≤0.05, ** p≤0.01, *** p≤0.001, **** p≤0.0001). n of field of views between 7 and 10 (as indicated by the dot plots), acquired over at least 2 biologically and technically independent experiments. Center of the box plots represents the median, hinges include first and third quartiles, and whiskers extend up to the smallest/largest value included in 1.5-fold the interquartile range (IQR).

c, Pearson correlation plot (two-sided) of the $\log_2$ expression of the neuropil : somata ratios of mRNA levels measured by FISH and by RNAseq (data sourced from [1]). Shaded area represents the 95% confidence interval.

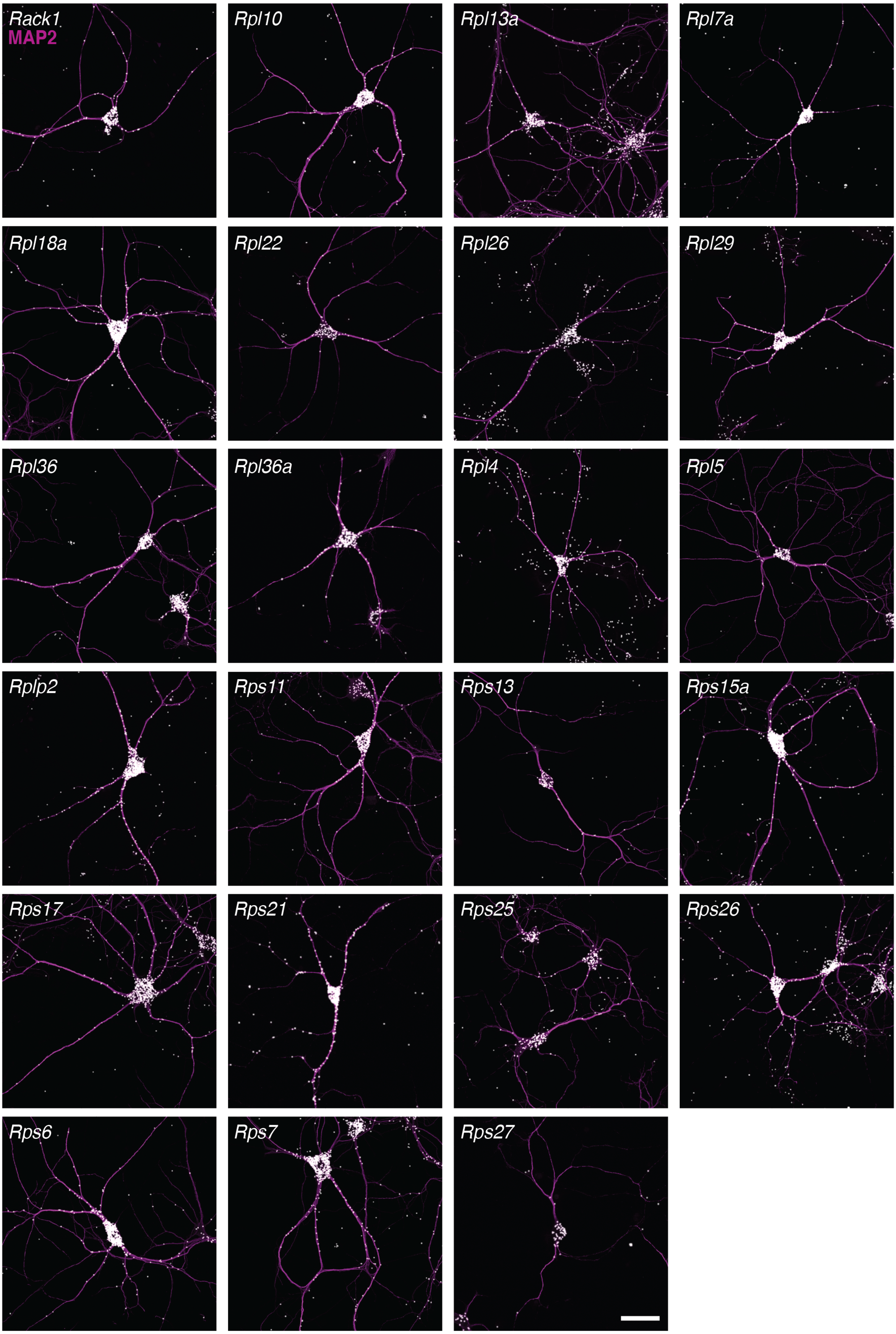

Supplementary Figure 2

Supplementary Figure 2: Detection of RP mRNAs in the dendrites of cultured hippocampal neurons.

FISH detection of indicated RP mRNAs (magenta MAP2, white FISH) in cultured hippocampal neurons Scale bar = 50 μm. Analysis for these data is shown in Fig. 1f. Representative images from n≥6 field of views.

a

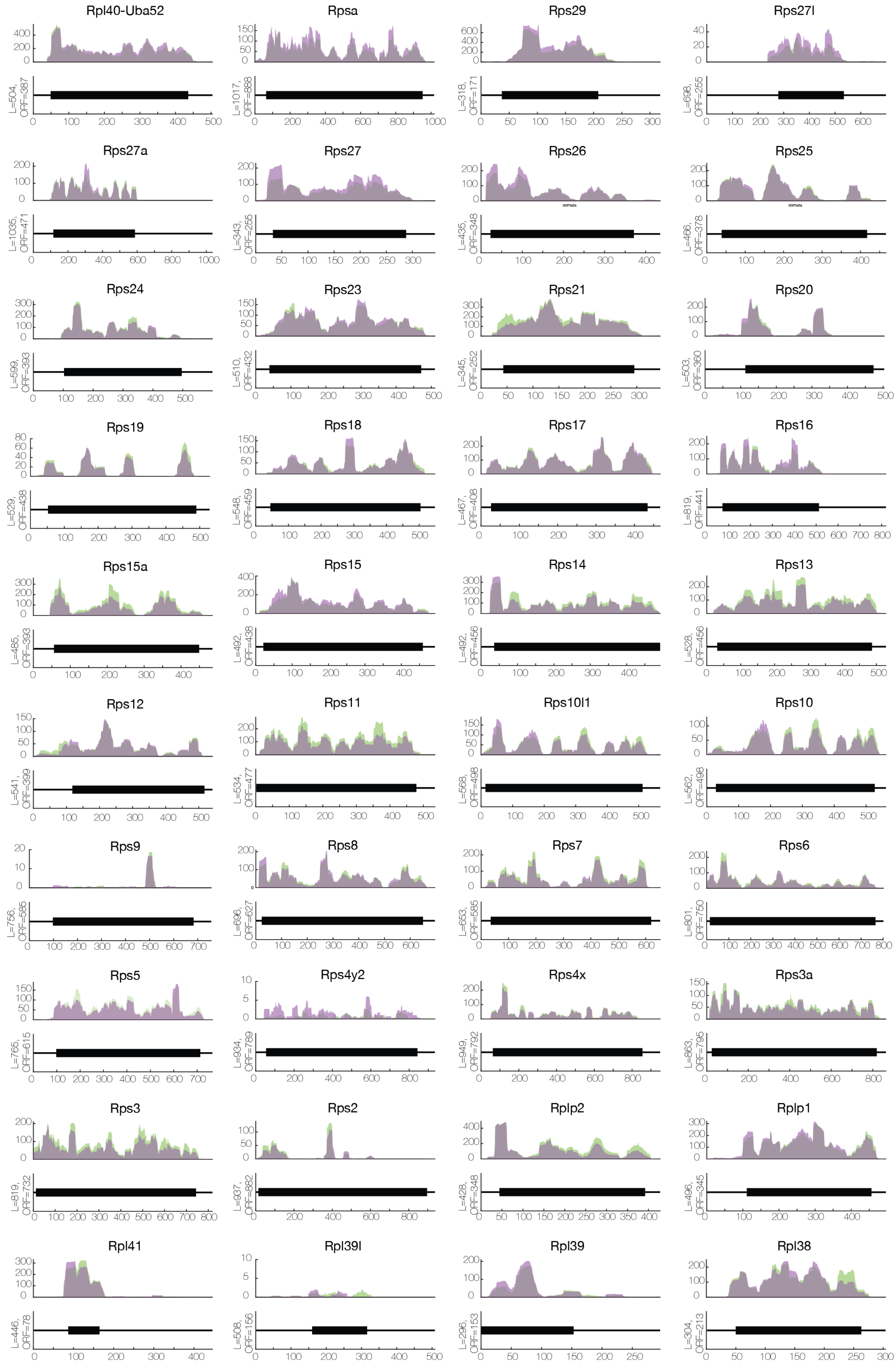

Supplementary Figure 3

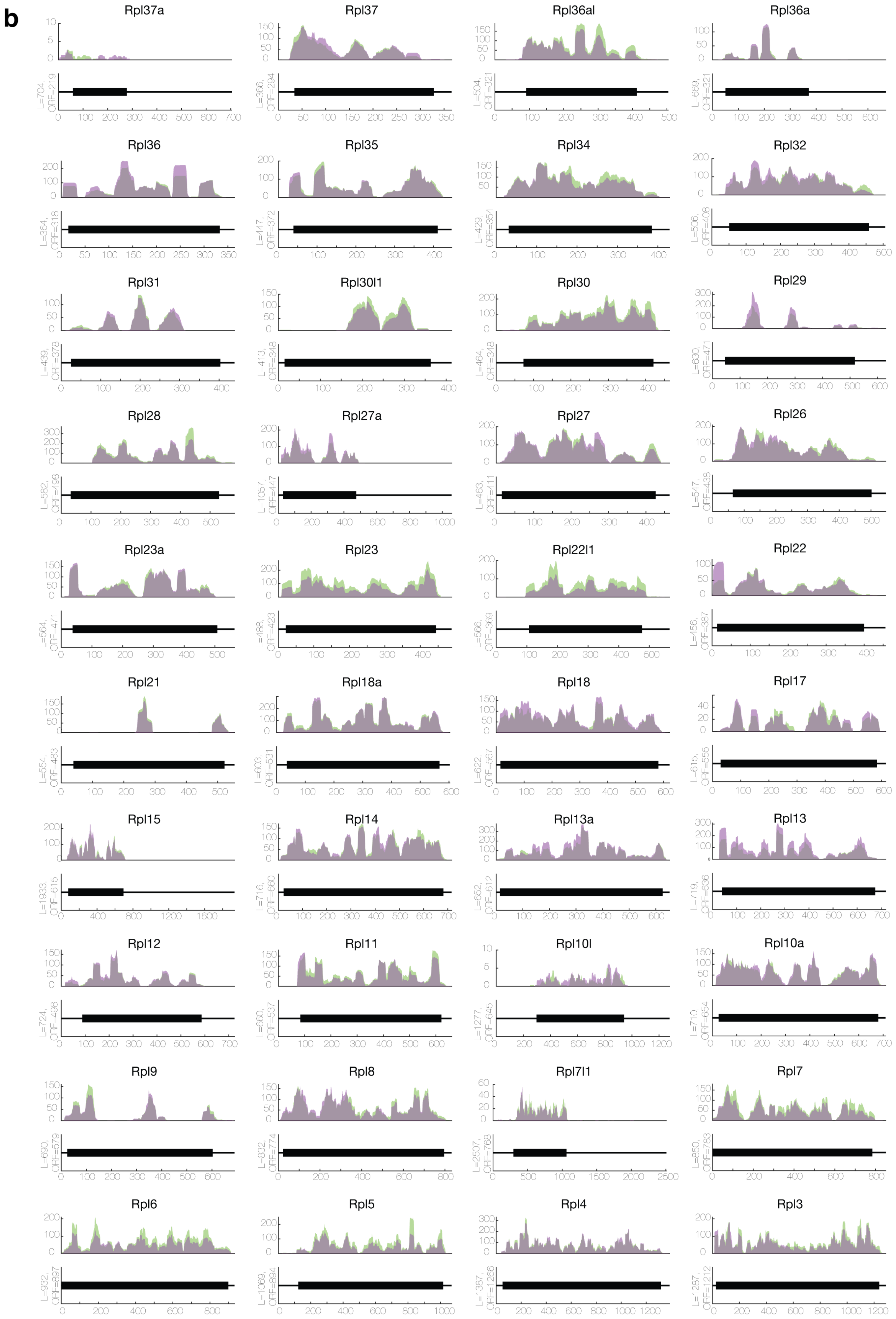
b
Rpl37a
Rpl37
Rpl36al
Rpl36a
Rpl36
Rpl35
Rpl34
Rpl32
Rpl31
Rpl30l1
Rpl30
Rpl29
Rpl28
Rpl27a
Rpl27
Rpl26
Rpl23a
Rpl23
Rpl22l1
Rpl22
Rpl21
Rpl18a
Rpl18
Rpl17
Rpl15
Rpl14
Rpl13a
Rpl13
Rpl12
Rpl11
Rpl10l
Rpl10a
Rpl9
Rpl8
Rpl7l1
Rpl7
Rpl6
Rpl5
Rpl4
Rpl3

Supplementary Figure 3: Ribosome footprint coverage of RP mRNAs in rat hippocampal slices.

a -b, Ribosome footprint coverage of RP mRNAs from the somata and neuropil of hippocampal slices[1]. Shown are the number of reads throughout the open reading frame (black box) from the somata-enriched fraction (green) or the neuropil-enriched fraction (purple). Regions of overlap appear as grey. The periodicity of the reads was confirmed for both the neuropil and somata ribosome profiling data in the original paper[1].

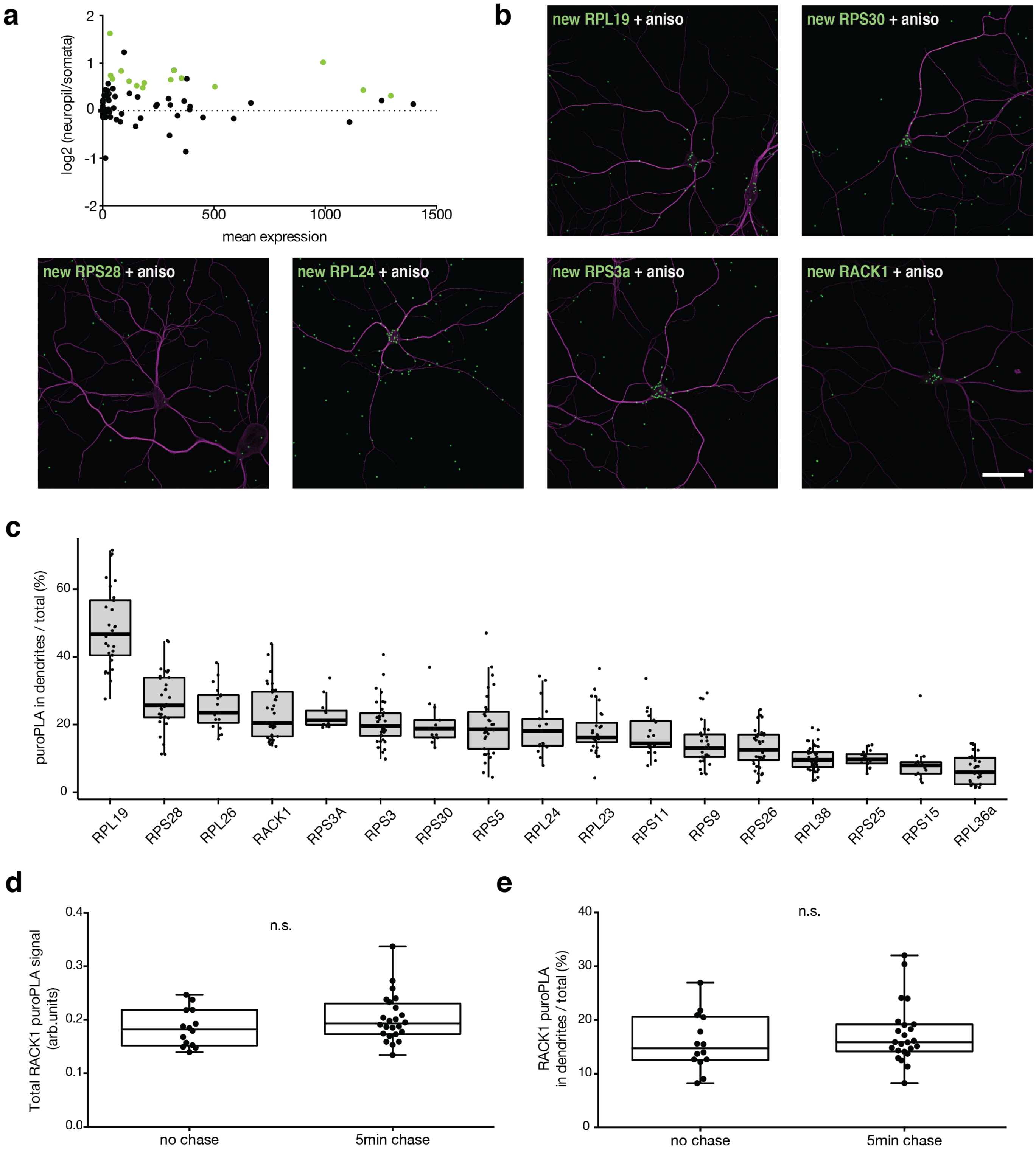

Supplementary Figure 4

Supplementary Figure 4: Quantification of dendritic RP mRNA translation.

a, Log_2 expression of the neuropil : somata ratios of RP footprints (data and statistical testing from [1]). RP mRNAs with a significant (adjusted p-value < 0.05, DESeq2) translation increase between the two compartments are highlighted in green. No transcript exhibited a somata-enhanced translation whereas 15 RP mRNAs showed a neuropil-enhanced translation.

b, Puro-PLA detection of nascent RPs in dendrites (magenta MAP2, green newly synthesized RP) in the presence of protein synthesis inhibitor anisomycin (compare to the images shown in Fig. 2b). Scale bar = 50 μm. Representative images from n≥4 field of views, acquired over ≥2 biologically and technically independent experiments,

c, Percentage of nascent RP signal in dendrites over total detected in individual neurons. Proteins are ranked according to their mean values. Center of the box plots represents the median, hinges include first and third quartiles, and whiskers extend up to the smallest/largest value included in 1.5-fold the interquartile range (IQR). n of cells between 13 and 40 (as indicated by the dot plots).

d-e, Total levels of nascent RACK1 in a neuron (**d**) or percentage of the signal in dendrites (**e**), immediately (no chase) or 5 min after (chase) labeling. Unpaired *t* test, two-tailed, n.s. p>0.5 (**e**, p=0.3992; **f**, p=0.1959). Center of the box plots represents the median, hinges include first and third quartiles, and whiskers extend up to the smallest/largest value. n of cells between 14 and 24 (as indicated by the dot plots), over at least 2 independent experiments.

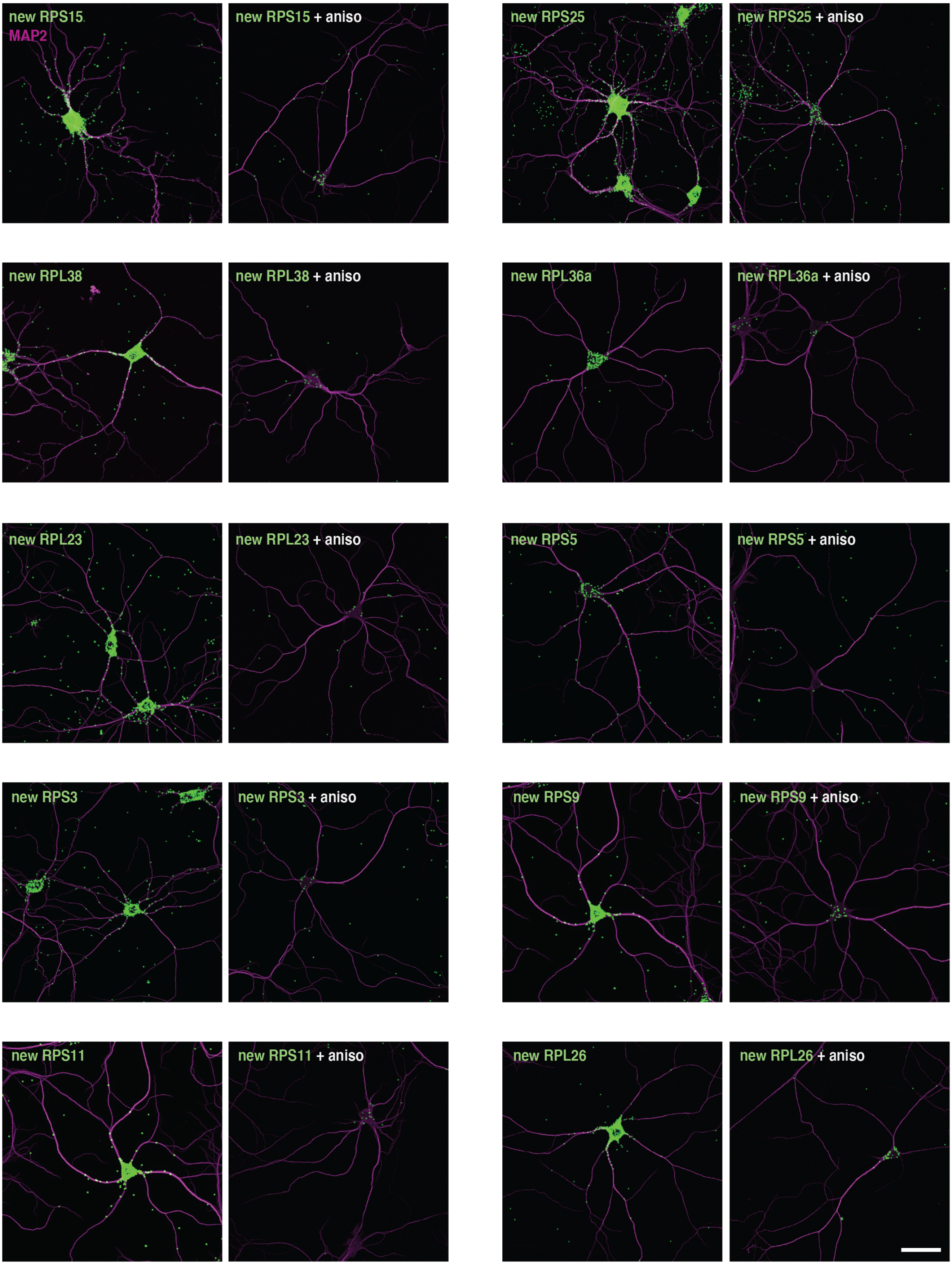

Supplementary Figure 5

Supplementary Figure 5: Detection of nascent RPs in dendrites of cultured hippocampal neurons.

Detection of nascent RPs (green) in dendrites (magenta for MAP2 immunostaining) of cultured hippocampal neurons using Puro-PLA [2]. Nascent proteins were labeled with puromycin (5 min), in the absence or presence (as indicated) of the protein synthesis inhibitor anisomycin (see methods). Scale bar = 50 μm. Representative images from n≥4 field of views, acquired over ≥2 biologically and technically independent experiments,

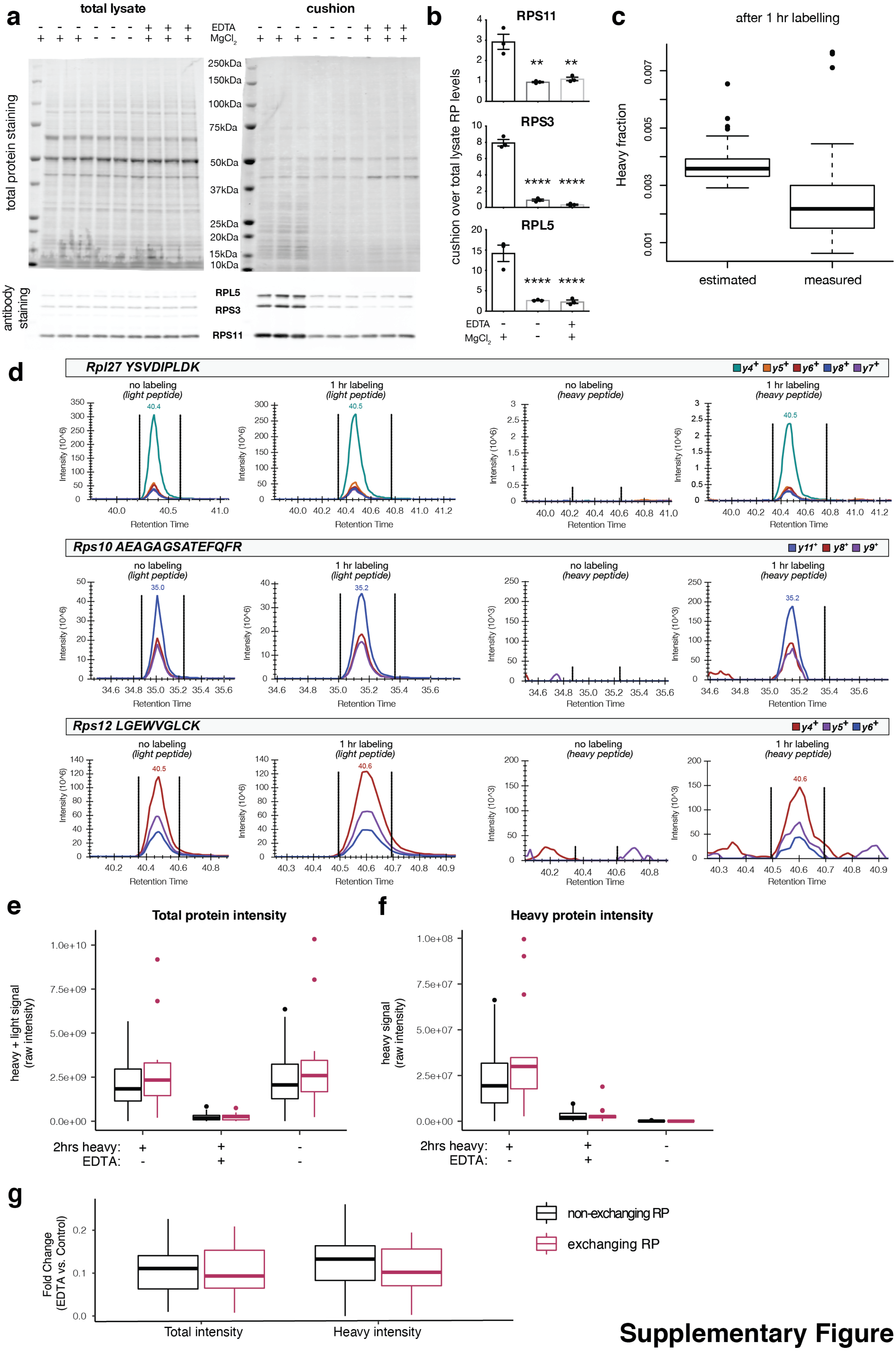

Supplementary Figure 6

Supplementary Figure 6: Quality controls for ribosome purification and mass spec detection of newly synthesized RPs.

a, Total protein and Western blot analysis of total lysates (left panel) or cushion samples (right panel) from three biological replicates. Cushion was performed under control conditions or in the absence of magnesium or in the presence of the chelating agent EDTA, as indicated. First lane contains molecular weight ladder. The same percentage of volumes was loaded for lysates and for cushion samples. Total protein (top panel) or RPs of interest (bottom panel) were visualized.

b, Quantification of Supplementary Fig.6a. The presence of individual RPs was significantly impaired when the cushion was performed under conditions that disassemble monosomes and polysomes into free small and large subunits. Three biological replicates. Data are presented as mean ± SEM. One-way ANOVA, with Dunnett's multiple comparisons test. ** $p \leq 0.01$, **** $p \leq 0.0001$

c, Fraction of labeled peptides (H/(H+L)) for ribosomal proteins (n=71), after 1 hr of labeling with heavy amino acids, estimated based on the half-life quantified by Dörrbaum et al. [3] or measured in our data set. Center of the box plots represents the median, hinges include first and third quartiles, and whiskers extend up to the smallest/largest value included in 1.5-fold the interquartile range (IQR). Outlying data points are presented as dots.

d, Examples of the traces for heavy or light peptides measured by Mass Spectrometry with Parallel Reaction Monitoring (see methods), after 1 hr of labeling or not, as indicated. Representative peptides from the indicated RPs of high (top panel), medium (middle panel) or low (bottom panel) abundance were chosen.

e, Box plot of the total (heavy + light) protein intensity of purified RPs after sucrose cushion in the presence or absence of EDTA, with or without a 2 hours incubation with heavy amino acids, as indicated by the x axis. As also shown by Western Blot in Extended Data Fig. 6a-b, the RPs signal is sensitive to the disassembly of translating ribosomes by EDTA, for both exchanging RPs (in purple) and non-exchanging RPs (in black). Center of the box plots represents the median, hinges include first and third quartiles, and whiskers extend up to the smallest/largest value included in 1.5-fold the interquartile range (IQR). Outlying data points are presented as dots. The value for each protein is the average of 3 biological replicates. Source data are provided as a Source Data file.

f, Box plot of the heavy protein intensity of purified RPs after sucrose cushion in the presence or absence of EDTA, with or without a 2 hours incubation with heavy amino acids, as indicated by the x axis. The new RP signal was sensitive to the heavy amino acids incubation, as well as to the disassembly of translating

ribosomes by EDTA, for both exchanging (in purple) and non-exchanging RPs (in black), confirming the specificity of our labeling for new assembled ribosomes. Center of the box plots represents the median, hinges include first and third quartiles, and whiskers extend up to the smallest/largest value included in 1.5-fold the interquartile range (IQR). Outlying data points are presented as dots. The value for each protein is the average of 3 biological replicates. Source data are provided as a Source Data file.

g, Box plot of the fold-change for the total (left panel) or heavy intensity (right panel) between EDTA and Control. The reduction with EDTA of the heavy signal was not significantly different from the reduction of the total signal, for both exchanging (in purple) and non-exchanging RPs (in black). Ordinary one-way ANOVA, $p>0.5$. Average of 3 biological replicates. Center of the box plots represents the median, hinges include first and third quartiles, and whiskers extend up to the smallest/largest value included in 1.5-fold the interquartile range (IQR). Outlying data points are presented as dots. Source data are provided as a Source Data file.

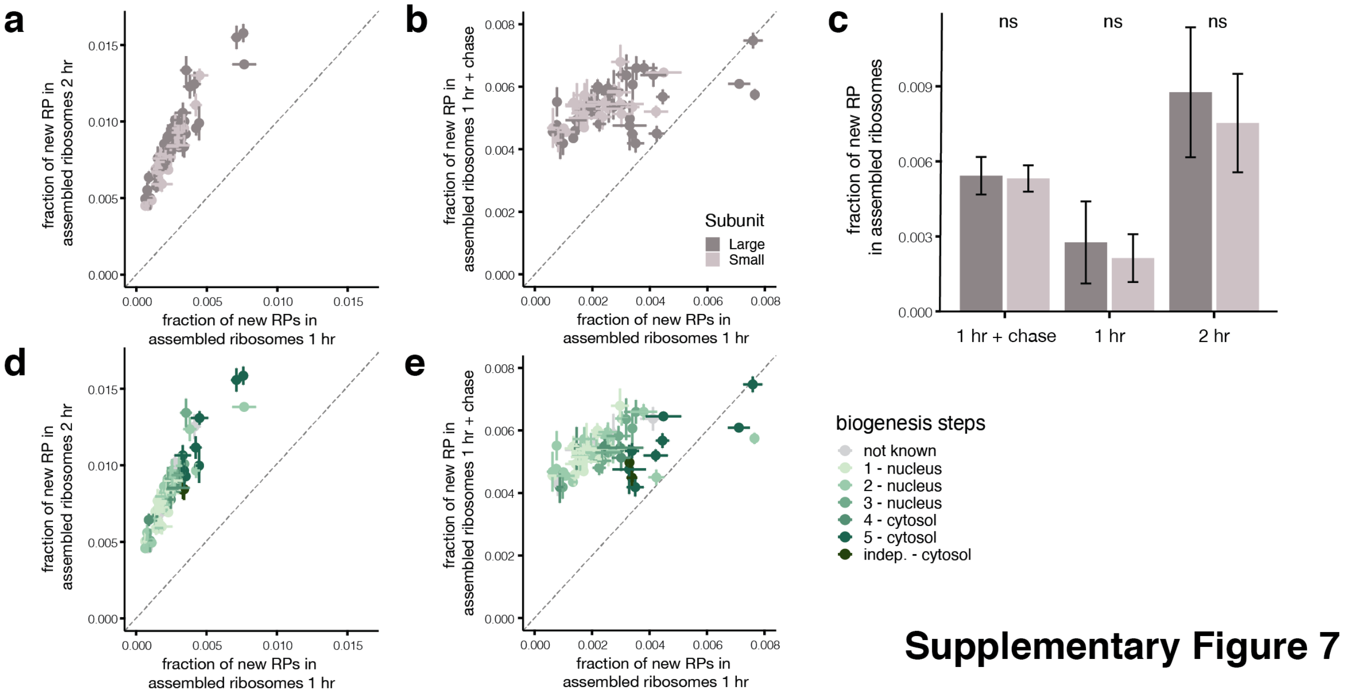

Supplementary Figure 7

Supplementary Figure 7: RPs incorporation dynamics according to subunits and canonical biogenesis steps.

a-b, Related to Fig. 3c-d. Scatterplots showing fraction of new RPs (H/(H+L)) in assembled ribosomes after different labeling conditions, as indicated in x- and y- axes. Points represent average +/- standard deviation of three biological replicates. Proteins are colored according to which ribosomal subunit they belong to (small subunit in light gray, large subunit in dark gray). Dashed line represents x = y.

c, Related to Fig. 3e-f. Mean +/- standard deviation of the fraction of new proteins (H/(H+L)) in assembled ribosomes for RPs of the same subunit (small subunit in light gray, n=45, large subunit in dark gray, n=33). Different labeling conditions are indicated on the x-axis. Wilcox test, n.s. p>0.05

d-e, Related to Fig. 3c-d. Scatterplots showing fraction of new RPs (H/(H+L)) in assembled ribosomes after different labeling conditions, as indicated by the x- and y- axes. Points represent average +/- standard deviation of three biological replicates. Proteins are colored according to which step they are known to incorporate into immature ribosomes during biogenesis (as reviewed in [4]). The darkest green color represents RPLP1 and RPLP2, whose incorporation is not linked to ribosome biogenesis. Dashed line represents x = y.

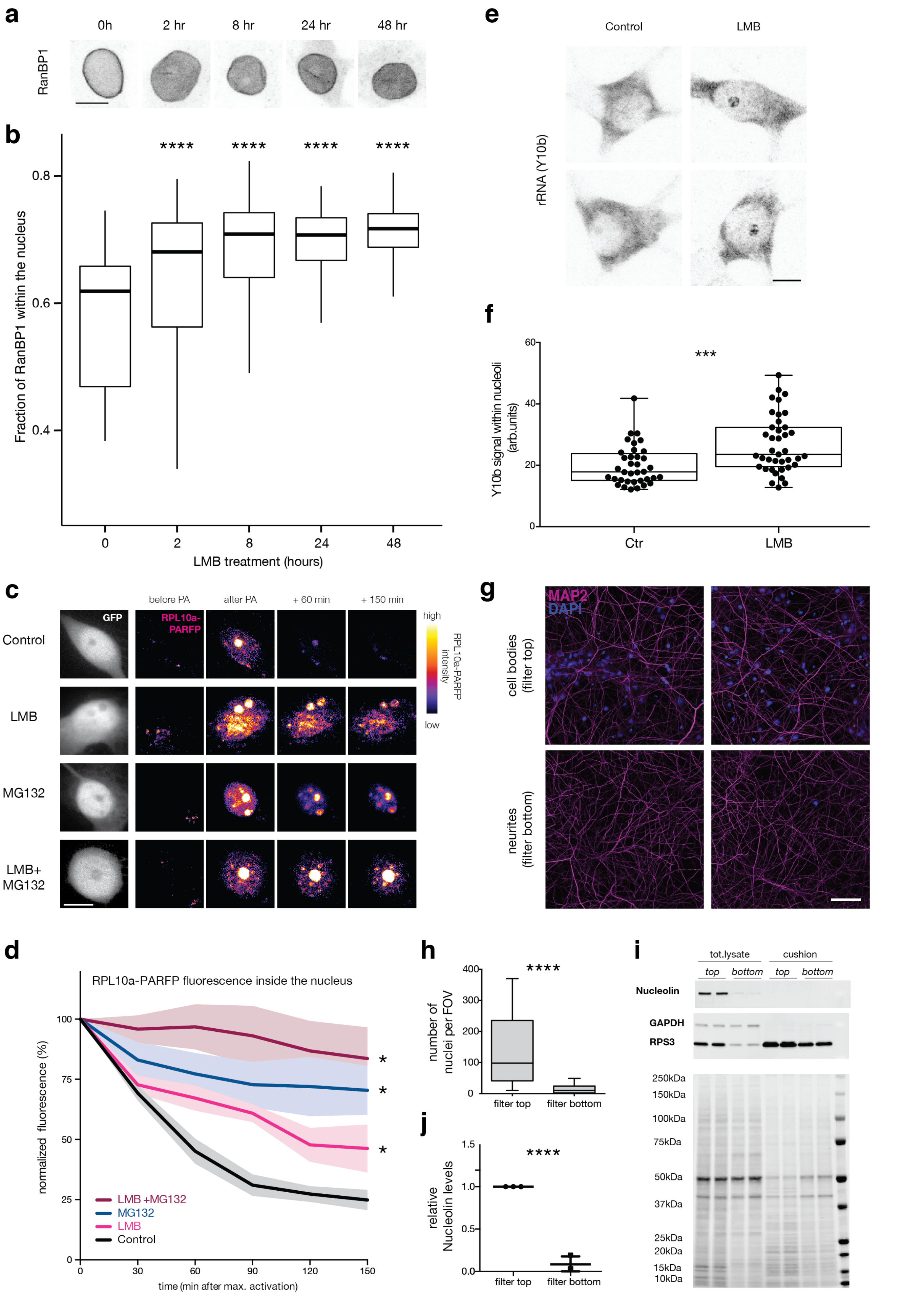

Supplementary Figure 8

Supplementary Figure 8: Leptomycin B (LMB) rapidly blocks CMR1-mediated nuclear export, and thus sequesters nascent ribosomes in the nucleus. Compartmentalized chambers allow for the enrichment of neurites.

a, Immunolabeling of RanBP1 in neuronal cell bodies at different durations of LMB incubation. RanBP1 is an accessory protein involved in the CRM1-mediated transport, known for its rapid shuffling between nucleus and cytoplasm. Scale bar = 10μm. Representative images from n≥170 cells, acquired over 2 biologically and technically independent experiments,

b, The fraction of RanBP1 signal within the nucleus significantly increases during LMB incubation at all time points tested (Wilcox test, **** $p < 0.001$). n between 170 and 400 cells, over 2 independent experiments. Center of the box plots represents the median, hinges include first and third quartiles, and whiskers extend up to the smallest/largest value included in 1.5-fold the interquartile range (IQR).

c, Representative images of neurons transfected with GFP (grey) and RPL10a-photo-activatable (PA)-RFP (fire color look-up), before and after photoactivation, under control conditions (first row) or in the presence of LMB to block nuclear export (second row), or MG132 to block proteasome-mediated degradation (third row), or both LMB and MG132 (fourth row). Scale bar = 10 μm. Representative images from n≥4 cells, acquired over 4 independent experiments,

d, Percentage of RPL10a-PARFP fluorescence inside the nucleus, normalized to the maximum nuclear intensity reached after photo-activation. Both the individual and combined inhibition of nuclear export and proteasomal degradation significantly slowed down the decay of RPL10a-PARFP signal in the nucleus (mean +/- SEM, repeated measures ANOVA, $p<0.01$, Dunnett's multiple comparisons test, $p<0.05$). n≥4 cells, acquired over ≥4 independent experiments,

e, Immunolabeling of rRNA (Y10b antibody) in neuronal cell bodies under control condition or after 2 days of LMB treatment. Scale bar = 10μm. Representative images from n≥35 cells, acquired over 3 biologically and technically independent experiments,

f, The intensity of Y10b signal inside nucleoli significantly increases after 2 days of LMB incubation (unpaired *t* test, two-tailed, $p<0.001$). Center of the box plots represents the median, hinges include first and third quartiles, and whiskers extend up to the smallest/largest value. n of cells between 35 and 39 (as indicated by the dot plots), over 3 independent experiments.

g, Representative images indicating the presence of dendrites (MAP2) and nuclei (DAPI) in the compartmentalized chamber. Top or bottom view of the filter, as indicated (scale bar 50 μm). Representative images from n≥48 field of views, acquired over 5 independent experiments,

h, Analysis of nuclear de-enrichment in the compartmentalized chambers. The number of nuclei per Field Of View (FOV) was dramatically decreased in neurite compartment (bottom). Five biological replicates, for a total of > 45 Fields of View per compartment. Unpaired *t* test, two-tailed, **** p≤0.0001. Center of the box plots represents the median, hinges include first and third quartiles, and whiskers extend up to the smallest/largest value. n of FOV between 48 and 53 (as indicated by the dot plots), over 5 independent experiments.

i, Total protein and Western blot analysis of lysates or cushion samples from somata + neurites (top) or neurites (bottom) compartments, as indicated. Last lane contains molecular weight ladder. Total protein (bottom panel) or proteins of interest (bottom panel) were visualized: Nucleolin as a nuclear marker, GAPDH as a cytosolic marker, RPS3 as a ribosome marker.

j, Quantification of protein analysis. The level of Nucleolin detected in the somata + neurites (top) fraction was significantly higher than that observed in the neurite (bottom) fraction. Three biological replicates. Unpaired *t* test, two-tailed, **** p≤0.0001. Data are presented as mean ± SD.

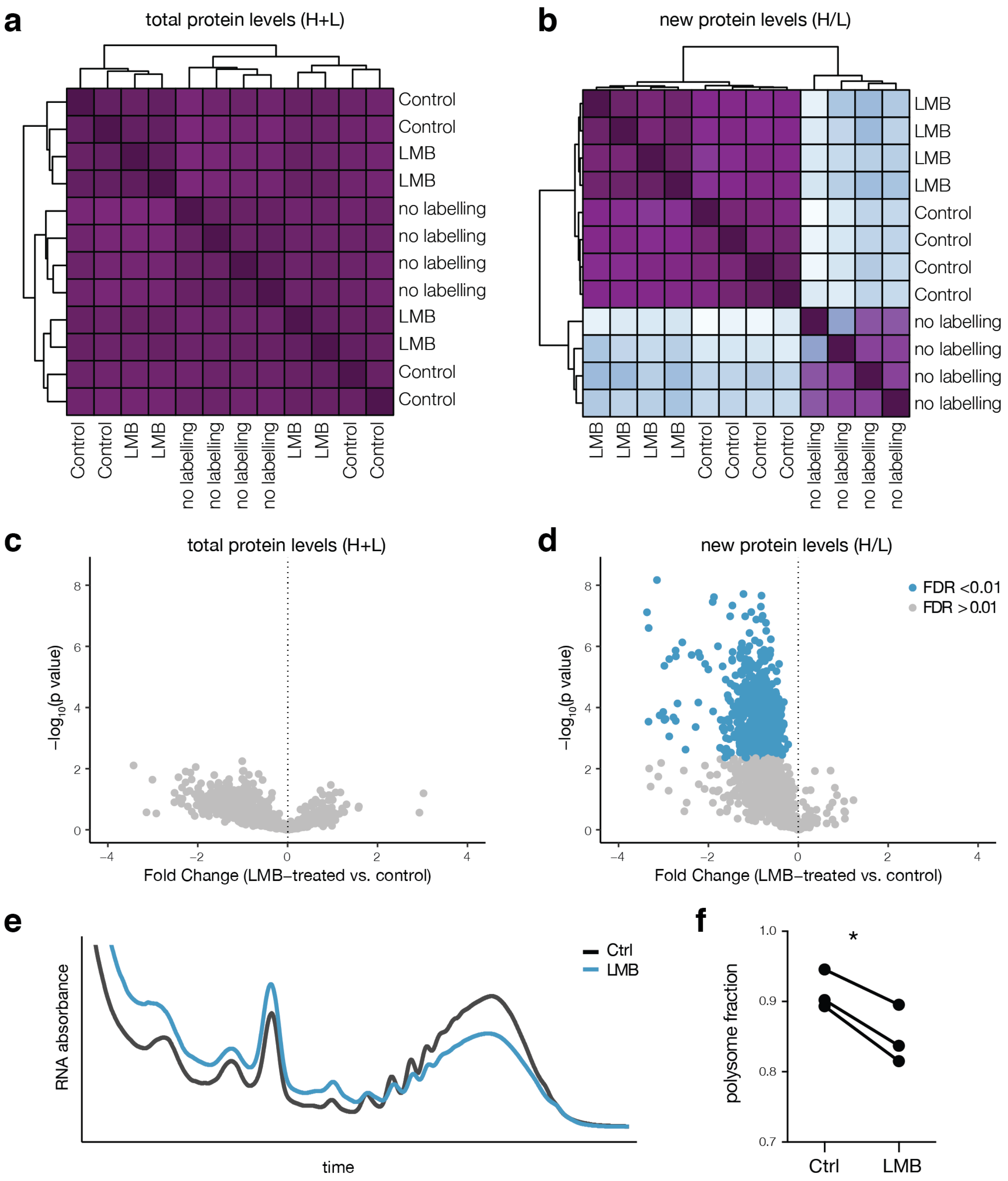

Supplementary Figure 9

Supplementary Figure 9: LMB treatment does not affect the total cellular protein levels but reduces the population of new proteins.

a-b, Hierarchical clustering of biological replicates (see methods) according to the similarity of total (log_2 of H+L, **A**) or new (log_2 of the H/L ratio, **B**) protein levels in lysates from control, LMB-treated or unlabeled neurons. Cells are color coded according to pairwise Pearson correlations. Biological replicates of the same labeling condition cluster together only when comparing the fraction of newly synthesized proteins.

c-d, Volcano plots of significantly regulated proteins (blue, FDR<0.01) in total lysates, comparing control and LMB-treated neurons. Nuclear export inhibition via LMB does not affect total protein levels (log_2 of H+L, **C**), but results in a general decrease in newly synthesized proteins (**D**). Dashed line represents Fold Change = 0. Unpaired, two-sided t-test with Welch correction on rows. Benjamini-Hochberg correction for multiple testing.

e, Representative polysome profiles from control (black) or LMB-treated (blue) neurons.

f, Quantification of the polysome fraction from control and LMB-treated neurons. Ribosome biogenesis inhibition via LMB resulted in a decrease in protein synthesis. Paired *t* test, two-tailed, * p=0.0156. Three biological replicates.

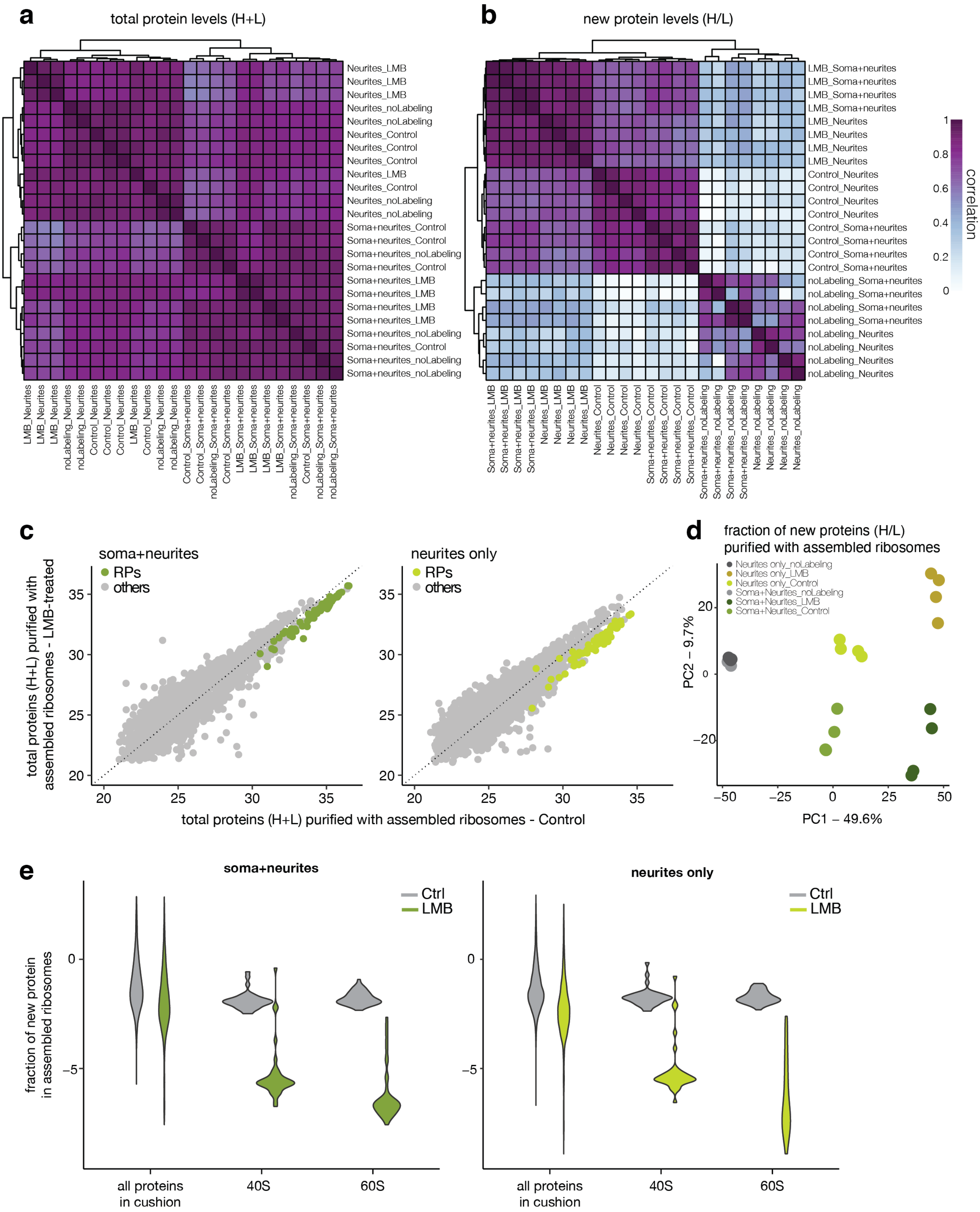

Supplementary Figure 10

Supplementary Figure 10: LMB treatment results in fewer new ribosomes.

a-b, Hierarchical clustering of biological replicates (see methods) according to similarity of total (log_2 of H+L, **a**) or new (log_2 of the H/L ratio, **b**) levels of proteins co-purified with assembled ribosomes from cell bodies or neurites of control, LMB-treated or unlabeled neurons. Cells are color coded according to pairwise Pearson correlations (two-sided). When comparing total protein levels, the unsupervised clustering segregates samples according to compartment but not according to labeling condition (**a**). When comparing the fraction of newly synthesized proteins instead, biological replicates of the same labeling condition and from the same compartment are successfully clustered together (**b**).

c,. Scatterplots showing total levels of protein (log_2 of H+L) co-purified with assembled ribosomes from cell bodies (left panel) or neurites (right panel) of control or LMB-treated neurons. Ribosomal proteins are indicated in green. Average of four biological replicates. Dashed line represents x = y.

d, PCA analysis showing similarities across new protein levels co-purified with assembled ribosomes from cell bodies or neurites of control, LMB-treated or unlabeled neurons.

e, Levels of new proteins (log_2 of the H/L ratio) co-purified with assembled ribosomes from cell bodies (left panel) or neurites (right panel) of control (grey) or LMB-treated (green) neurons. Ribosomal proteins from either small (40S) or large (60S) subunit are grouped as indicated in the x-axis. Average of four biological replicates.

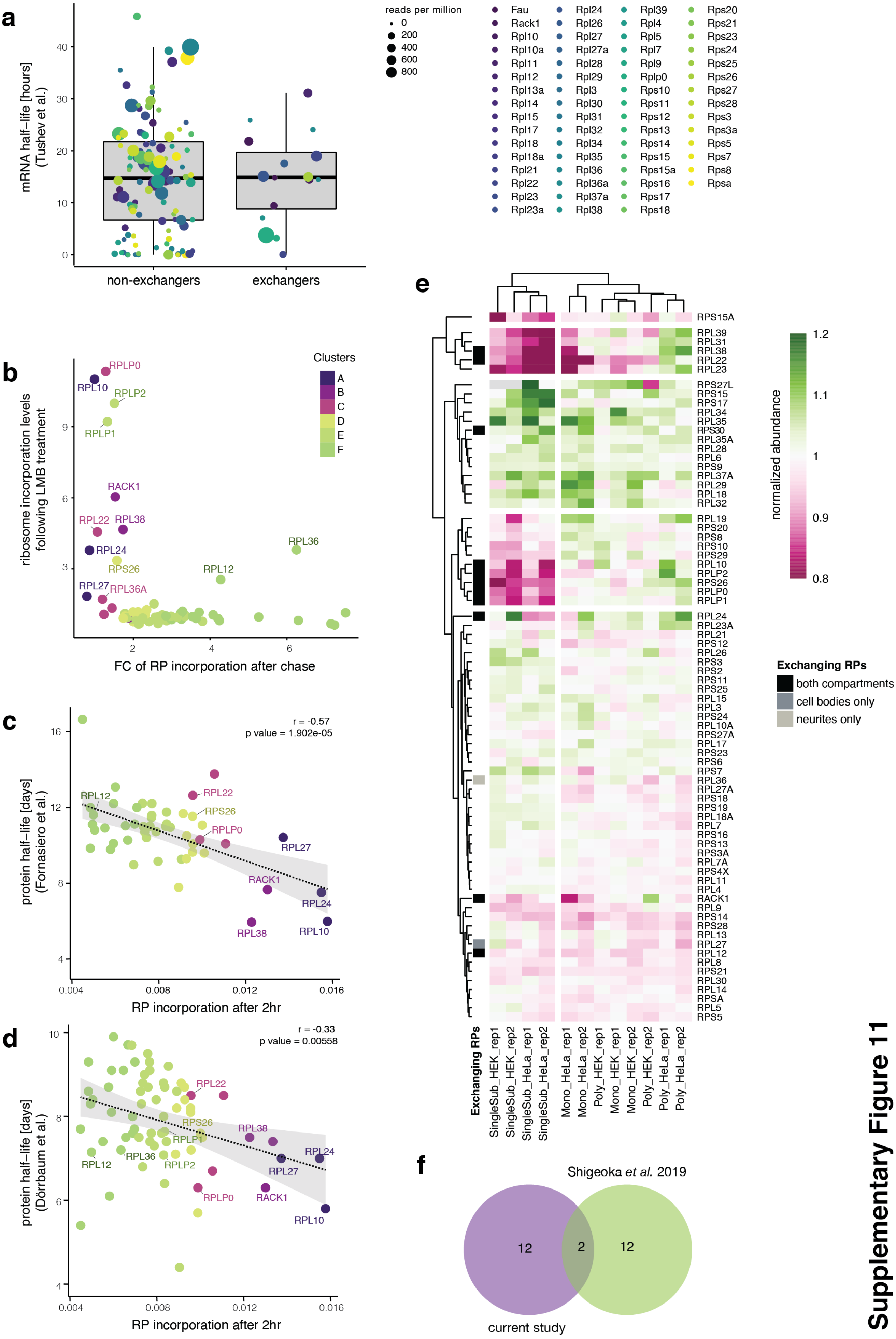

Supplementary Figure 11

Supplementary Figure 11: Exchanging RPs tend to be short-lived and sub-stoichiometric in single subunits.

a, Boxplot showing mRNA half-life, as measured by [5], of transcripts coding for exchanging (n=16) or non-exchanging (n=112) RPs, as indicated by the x axis. Transcript isoforms of the same gene are shown in the same color, as indicated in the legend. The size of each dot is proportional to the transcript expression level in cultured neurons (measured as reads per million). Center of the box plots represents the median, hinges include first and third quartiles, and whiskers extend up to the smallest/largest value included in 1.5-fold the interquartile range (IQR).

b, Scatterplot showing the fold change of RP incorporation after a chase (x-axis, relative to Fig. 3d) and the incorporation levels after LMB treatment (y-axis, average between compartments from Fig. 4d). Ribosomal proteins are colored according to the clusters identified in Fig. 3b. Exchanging RPs are indicated by name.

c-d, Scatterplots showing the incorporation of new RPs after 2 hr of labeling (x-axis, as in Fig. 3) and their half-life (y-axis) as measured in intact cortical tissue ([6], **c**) or in cultured hippocampal neurons ([3], **d**). Proteins are colored according to the clusters identified in Fig. 3b. Exchanging RPs are indicated by name. Dashed line represents the regression line and the 95% confidence interval is shown in grey. Pearson correlation (two-sided).

e, Heatmap showing RP expression levels across different ribosomal conformations (selected fractions within polysome profiling gradients) in HEK and HeLa cells, as measured using MS by [7]. Pseudocells (protein levels normalized to the median of the corresponding ribosomal subunit, see Methods) are ordered using unsupervised clustering, both for columns (biological replicate of each condition) and rows (individual ribosomal protein). The exchanging RPs in both compartments are shown in black, those exchanging only in the compartment with cell bodies are shown in dark grey, and those exchanging only in neurites are shown in light grey (as identified in Fig. 4d). SingleSub = single ribosomal subunits (large or small). Mono = monosome. Poly = polysome.

f, Venn diagram showing the overlap between the exchanging RPs identified in the current study (purple) and the nascent RPs detected in axonal ribosomes after removal of the cell body in a recent study ([8], green). Curiously, the two known positive controls of exchange, RPLP1 and P2, were not detected in [8].

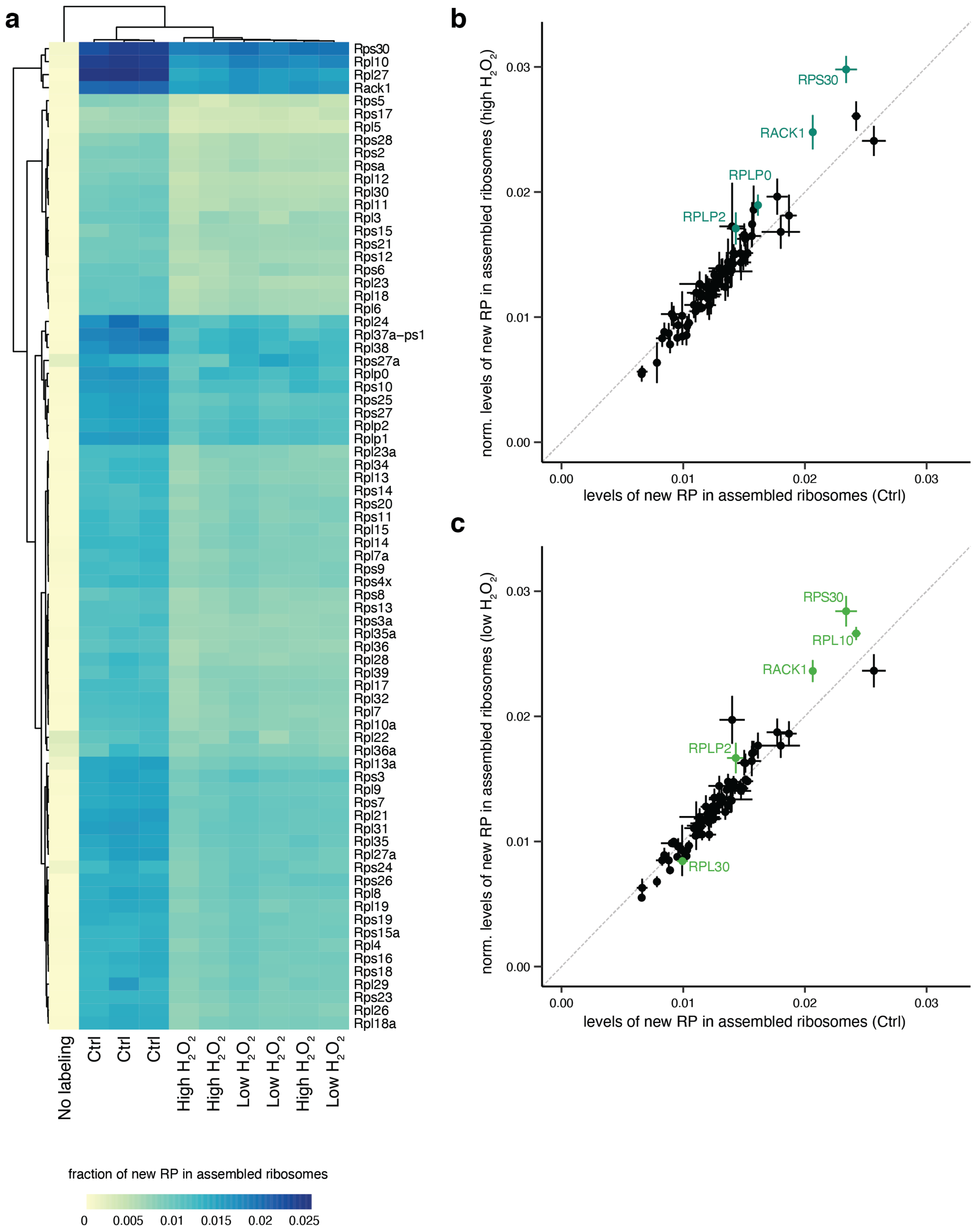

Supplementary Figure 12

Supplementary Figure 12: Effect of oxidative stress on RPs incorporation into assembled ribosomes.

a, Heatmap showing for each RP the fraction of new proteins (H/(H+L)) incorporated into assembled ribosomes. Pseudocells (median of peptides obtained per individual protein) are ordered according to unsupervised clustering, both for columns (biological replicate of each condition) and rows (individual ribosomal protein). Experimental conditions of the labeling are indicated at the bottom. Source data are provided as a Source Data file.

b-c, Scatterplots showing the fraction of new RPs (H/(H+L)) in assembled ribosomes after the different labeling conditions, as indicated by x- and y- axes. To correct for the general decrease in protein synthesis with stress, the values in the H_2O_2 samples were normalized over the mean fold change. Points represent average +/- standard deviation of three biological replicates. Proteins are colored according to the significance (green, FDR<0.01) calculated as in Fig. 5b. Dashed line represents x=y. See methods for more information on statistical testing. Source data are provided as a Source Data file.

Supplementary Table 1: FISH Probe set.

Protein name	Gene symbol	ViewRNA ISH Cell Assay Probes set (ThermoFisher, QVC0001)
CamKIIa	CaMK2a	VC1-14332
Histone3	H3f3b	VC6-11342
RACK1	Gnbl2l1	VC1-3061703
RPL10/uL16	Rpl10	VC1-20759
RPL13a/uL13	Rpl13a	VC6-3229187
RPL18a/eL20	Rpl18a	VC6-3234577
RPL19/eL19	Rpl19	VC1-17134
RPL22/eL22	Rpl22	VC1-3061648
RPL26/uL24	Rpl26	VC1-20756
RPL29/eL29	Rpl29	VC1-20757
RPL36/eL36	Rpl36	VC1-3060810
RPL36A/eL42	Rpl36a	VC1-3065218
RPL38/eL38	Rpl38	VC1-3077564
RPL4/uL4	Rpl4	VC1-20758
RPL5/uL18	Rpl5	VC6-3231617
RPL7a/eL8	Rpl7a	VC6-3236240
RPLP0/uL10	Rplp0	VC1-10192
RPLP1/P1	Rplp1	VC1-3062658
RPLP2/P2	Rplp2	VC1-3062659
RPS11/uS17	Rps11	VC1-3061654
RPS13/uS15	Rps13	VC1-20760
RPS15a/uS8	Rps15a	VC1-3062503
RPS17/eS17	Rps17	VC1-3060264
RPS21/eS21	Rps21	VC1-3061655
RPS25/eS25	Rps25	VC1-3062623
RPS26/eS26	Rps26	VC1-3060146
RPS27/eS27	Rps27	VC1-3062104
RPS29/uS14	Rps29	VC1-10529
RPS30/eS30	Fau	VC1-3060571
RPS6/eS6	Rps6	VC1-3060274
RPS7/eS7	Rps7	VC6-3060244

Supplementary Table 2: Antibody list.

Target	Company	Identifier	Dilution
GAPDH	abcam	ab8245	1:2500 (WB)
MAP2	SYSY	188004	1:2000 or 1:1000 (IF)
Nucleolin	abcam	ab31163	1:10000 (IF) 1:2000 (WB)
puromycin	Kerafast	EQ0001	1:3500 (IF)
puromycin	CRB-cambridge	RANV10RbE76	1:200 (IF)
RACK1	abcam	ab62735	1:200 (IF)
RanBP1	abcam	ab97659	1:200 (IF)
RPL19/eL19	abcam	ab224592	1:200 (IF)
RPL23/uL14	Proteintech	16086-1-AP	1:200 (IF)
RPL26/uL24	SIGMA	R0655	1:1000 (IF)
RPL36A/eL42	Santa Cruz	sc-100831	1:200 (IF)
RPL38/eL38	Bethyl	A305-412A	1:2000 (IF)
RPL5/uL18	abcam	ab186857	1:1000 (WB)
RPS11/uS17	Bethyl	A303-936A	1:1000 (IF) 1:1000 (WB)
RPS15/uS19	abcam	ab154936	1:500 (IF)
RPS25/eS25	ThermoFisher	PA5-56865	1:1000 (IF)
RPS26/eS26	abcam	ab229571	1:50 (IF)
RPS28/eS28	abcam	ab133963	1:50 (IF)
RPS3/uS3	Bethyl	A303-840A-M	1:1000 (WB) 1:100 (IF)
RPS30/eS30	abcam	ab239073	1:500 (IF)
RPS3A/eS1	Bethyl	A305-001A	1:1000 (IF)
RPS5/uS7	Bethyl	A304-010A-M	1:250 (IF)
RPS9/uS4	Bethyl	A303-946A-M	1:400 (IF)
Y10b	abcam	ab171119	1:1000 (IF)
Goat anti-guinea pig Dylight405	Jackson ImmunoResearch	106-475-003	1:1000 (IF)
Goat anti-guinea pig-Alexa488	ThermoFisher	A11073	1:1000 (IF)
Goat anti-mouse-Alexa594	ThermoFisher	A11005	1:1000 (IF)
Goat anti-mouse-Alexa488	ThermoFisher	A11001	1:1000 (IF)
Goat anti-rabbit-Alexa594	ThermoFisher	A11037	1:1000 (IF)
Goat anti- rabbit-Alexa488	ThermoFisher	A11008	1:1000 (IF)

Supplementary References.

1. Biever, A. *et al.* Monosomes actively translate synaptic mRNAs in neuronal processes. *Science* **367,** eaay4991–16 (2020).
2. tom Dieck, S. *et al.* Direct visualization of newly synthesized target proteins in situ. *Nature Methods* **12,** 411–414 (2015).
3. Dörrbaum, A. R., Kochen, L., Langer, J. D. & Schuman, E. M. Local and global influences on protein turnover in neurons and glia. *eLife* **7,** 489 (2018).
4. la Cruz, de, J., Karbstein, K. & Woolford, J. L., Jr. Functions of Ribosomal Proteins in Assembly of Eukaryotic Ribosomes In Vivo. *Annu. Rev. Biochem.* **84,** 93–129 (2015).
5. Tushev, G. *et al.* Alternative 3' UTRs Modify the Localization, Regulatory Potential, Stability, and Plasticity of mRNAs in Neuronal Compartments. *Neuron* **98,** 495–511.e6 (2018).
6. Fornasiero, E. F. *et al.* Precisely measured protein lifetimes in the mouse brain reveal differences across tissues and subcellular fractions. *Nat Comms* **9,** 4230 (2018).
7. Imami, K. *et al.* Phosphorylation of the Ribosomal Protein RPL12/ uL11 Affects Translation during Mitosis. *Mol Cell* **72,** 84–98.e9 (2018).
8. Shigeoka, T. *et al.* On-Site Ribosome Remodeling by Locally Synthesized Ribosomal Proteins in Axons. *CellReports* **29,** 3605–3619.e10 (2019).
