# Supplementary material for: Neuronal ribosomes exhibit dynamic and context-dependent exchange of ribosomal proteins
Source: Nat Commun. 2021 Oct 21;12:6127. doi: 10.1038/s41467-021-26365-x (PMC8531293; doi:10.1038/s41467-021-26365-x)
Supplement: Supplementary file 3 — Description of Additional Supplementary Files [file 41467_2021_26365_MOESM3_ESM.pdf]

Description of Additional Supplementary Files

File Name: Supplementary Data 1
Description: Detection of Ribosomal Proteins mRNAs in previously reported studies (relative to Fig. 1).

File Name: Supplementary Data 2
Description: Detailed summary of the parameters used for LC+MS.

File Name: Supplementary Data 3
Description: Detailed summary of the settings used for MaxQuant.
