# Supplementary material for: Neuronal ribosomes exhibit dynamic and context-dependent exchange of ribosomal proteins
Source: Nat Commun. 2021 Oct 21;12:6127. doi: 10.1038/s41467-021-26365-x (PMC8531293; doi:10.1038/s41467-021-26365-x)
Supplement: Supplementary file 7 — Reporting Summary [file 41467_2021_26365_MOESM7_ESM.pdf]

nature research

Corresponding author(s): Erin M. Schuman

Last updated by author(s): Sep 20, 2021

Reporting Summary

Nature Research wishes to improve the reproducibility of the work that we publish. This form provides structure for consistency and transparency in reporting. For further information on Nature Research policies, see our Editorial Policies and the Editorial Policy Checklist.

Statistics

For all statistical analyses, confirm that the following items are present in the figure legend, table legend, main text, or Methods section.

n/a	Confirmed	
☐	☒	The exact sample size (*n*) for each experimental group/condition, given as a discrete number and unit of measurement
☐	☒	A statement on whether measurements were taken from distinct samples or whether the same sample was measured repeatedly
☐	☒	The statistical test(s) used AND whether they are one- or two-sided<br>*Only common tests should be described solely by name; describe more complex techniques in the Methods section.*
☒	☐	A description of all covariates tested
☐	☒	A description of any assumptions or corrections, such as tests of normality and adjustment for multiple comparisons
☐	☒	A full description of the statistical parameters including central tendency (e.g. means) or other basic estimates (e.g. regression coefficient) AND variation (e.g. standard deviation) or associated estimates of uncertainty (e.g. confidence intervals)
☐	☒	For null hypothesis testing, the test statistic (e.g. *F*, *t*, *r*) with confidence intervals, effect sizes, degrees of freedom and *P* value noted<br>*Give P values as exact values whenever suitable.*
☒	☐	For Bayesian analysis, information on the choice of priors and Markov chain Monte Carlo settings
☐	☒	For hierarchical and complex designs, identification of the appropriate level for tests and full reporting of outcomes
☐	☒	Estimates of effect sizes (e.g. Cohen's *d*, Pearson's *r*), indicating how they were calculated

Our web collection on statistics for biologists contains articles on many of the points above.

Software and code

Policy information about availability of computer code

Data collection	See Materials and Methods for detailed descriptions.<br>For proteomics data set, MaxQuant (version 1.6.2.3 and 1.6.0.1; RRID:SCR_015753) and Skyline (version 20.1.0.155; RRID: SCR_014080) were used.<br>For images acquired at the inverted spinning disk confocal microscope (Zeiss 3i imaging systems; model CSU-X1), the software Slidebook (version 5.5.5; RRID:SCR_014300) was used.<br>For images acquired at the LSM780 confocal microscopy (Zeiss, version 14.0.0), the software ZEN 2.3 SP1 FP3 (version 14.0.23.201) was used.<br>For Western blot, images were acquired using LI-COR Image Studio Lite (version 3.0.30, RRID:SCR_013715).
Data analysis	See Materials and Methods for detailed descriptions.<br>Proteomics data sets were analyzed in RStudio (version 1.4.1106; RRID:SCR_000432). The pheatmap R-package (RRID: SCR_016418, https://CRAN.R-project.org/package=pheatmap) was used.<br>Images were analyzed in Fiji/ImageJ (version 2.1.0/1.53c; RRID:SCR_002285).<br>For the statistical analysis of same experiments (like Western Blot, SASA, immunofluorescence and outliers identification), GraphPad Prism (RRID:SCR_002798, version 9.1.0) was used.

For manuscripts utilizing custom algorithms or software that are central to the research but not yet described in published literature, software must be made available to editors and reviewers. We strongly encourage code deposition in a community repository (e.g. GitHub). See the Nature Research guidelines for submitting code & software for further information.

Data

Policy information about availability of data

All manuscripts must include a data availability statement. This statement should provide the following information, where applicable:

- Accession codes, unique identifiers, or web links for publicly available datasets
- A list of figures that have associated raw data
- A description of any restrictions on data availability

All MS data associated with this manuscript have been uploaded to the PRIDE repository (RRID:SCR_003411) and are available with the dataset identifier PXD026973.

Field-specific reporting

Please select the one below that is the best fit for your research. If you are not sure, read the appropriate sections before making your selection.

- [x] Life sciences
- [ ] Behavioural & social sciences
- [ ] Ecological, evolutionary & environmental sciences

For a reference copy of the document with all sections, see nature.com/documents/nr-reporting-summary-flat.pdf

Life sciences study design

All studies must disclose on these points even when the disclosure is negative.

Sample size	Sample size was not statistically predetermined, and is shown in the figures and figure legends.<br>For experiments with generally low variability between samples (for example Western Blots, qPCR, Mass Spectrometry, polysome profiling), sample size was chosen, in agreement with common practice in the field, between N=3 and N=5.<br>For experiments that rely on the quantification of single cells (for example Puro-PLA and FISH of cultured hippocampal neurons), where higher variability is observed, N≥6 was chosen.
Data exclusions	No sample was excluded.<br>For the targeted Mass Spectrometry data sets, peptides that did not pass quality filters (according to retention time, mass accuracy and library matches) were excluded.
Replication	Data measured by qPCR was generated in 3 technical replicates.<br>Data measured by DDA Mass Spectrometry was generated in 2 technical replicates.<br>All samples were measured in at least 3 biological replicates, as indicated in figures and figure legends.
Randomization	Samples were randomly allocated into experimental groups.
Blinding	Blinding was not performed in this study, as the quantification pipeline is streamlined and applied equally to all conditions.<br>As indicated in the manuscript, during imaging experiments that require manual selection of individual cells (for example Puro-PLA and FISH of cultured hippocampal neurons), cells were picked blind to the channel later used for quantification.

Reporting for specific materials, systems and methods

We require information from authors about some types of materials, experimental systems and methods used in many studies. Here, indicate whether each material, system or method listed is relevant to your study. If you are not sure if a list item applies to your research, read the appropriate section before selecting a response.

Materials & experimental systems

n/a	Involved in the study	
[ ]	[x]	Antibodies
[x]	[ ]	Eukaryotic cell lines
[x]	[ ]	Palaeontology and archaeology
[ ]	[x]	Animals and other organisms
[x]	[ ]	Human research participants
[x]	[ ]	Clinical data
[x]	[ ]	Dual use research of concern

Methods

n/a	Involved in the study	
[x]	[ ]	ChIP-seq
[x]	[ ]	Flow cytometry
[x]	[ ]	MRI-based neuroimaging

Antibodies

Antibodies used	Target Company Identifier<br>GAPDH abcam ab8245

MAP2 SYSY 188004
Nucleolin abcam ab31163
puromycin Kerafast EQ0001
puromycin CRB-cambridge RANV10RbE76
RACK1 abcam ab62735
RanBP1 abcam ab97659
RPL19/eL19 abcam ab224592
RPL23/uL14 Proteintech 16086-1-AP
RPL26/uL24 SIGMA R0655
RPL36A/eL42 Santa Cruz sc-100831
RPL38/eL38 Bethyl A305-412A
RPL5/uL18 abcam ab186857
RPS11/uS17 Bethyl A303-936A
RPS15/uS19 abcam ab154936
RPS25/eS25 ThermoFisher PA5-56865
RPS26/eS26 abcam ab229571
RPS28/eS28 abcam ab133963
RPS3/uS3 Bethyl A303-840A-M
RPS30/eS30 abcam ab239073
RPS3A/eS1 Bethyl A305-001A
RPS5/uS7 Bethyl A304-010A-M
RPS9/uS4 Bethyl A303-946A-M
Y10b abcam ab171119
Goat anti-guinea pig Dylight405 Jackson ImmunoResearch 106-475-003
Goat anti-guinea pig-Alexa488 ThermoFisher A11073
Goat anti-mouse-Alexa594 ThermoFisher A11005
Goat anti-mouse-Alexa488 ThermoFisher A11001
Goat anti-rabbit-Alexa594 ThermoFisher A11037
Goat anti-rabbit-Alexa488 ThermoFisher A11008
Donkey anti-guinea pig Cy5 Dianova 706-175-148

Validation

We used only commercially available antibodies, which are commonly used in the field. Validation information can be found on the website https://app.benchsci.com/

Animals and other organisms

Policy information about studies involving animals; ARRIVE guidelines recommended for reporting animal research

Laboratory animals

Animals were used only to generate primary neuronal cultures. For this purpose, Rattus norvegicus (Sprague-Dawley strain; Charles River Laboratories, RRID: RGD_734476), pups (postnatal day 0 to 2) of either sex were used.

Wild animals

No wild animal was used in this study.

Field-collected samples

They study did not involved any sample collected from the field.

Ethics oversight

Housing and sacrificing procedures complied with German national and international animal care policies and the guidelines issued by the Max Planck Society (DIRECTIVE 2010/63/EU; German animal welfare law; FELASA guidelines) and were approved by local authorities (Regierungspräsidium Darmstadt).

Note that full information on the approval of the study protocol must also be provided in the manuscript.
