# Supplementary material for: Neuronal ribosomes exhibit dynamic and context-dependent exchange of ribosomal proteins
Source: Nat Commun. 2021 Oct 21;12:6127. doi: 10.1038/s41467-021-26365-x (PMC8531293; doi:10.1038/s41467-021-26365-x)
Supplement: Supplementary file 2 — Peer Review File [file 41467_2021_26365_MOESM2_ESM.pdf]

nature portfolio

Peer Review File

Neuronal ribosomes exhibit dynamic and context-dependent exchange of ribosomal proteins.

Open Access This file is licensed under a Creative Commons Attribution 4.0 International License, which permits use, sharing, adaptation, distribution and reproduction in any medium or format, as long as you give appropriate credit to the original author(s) and the source, provide a link to the Creative Commons license, and indicate if changes were made. In the cases where the authors are anonymous, such as is the case for the reports of anonymous peer reviewers, author attribution should be to 'Anonymous Referee' followed by a clear attribution to the source work. The images or other third party material in this file are included in the article’s Creative Commons license, unless indicated otherwise in a credit line to the material. If material is not included in the article’s Creative Commons license and your intended use is not permitted by statutory regulation or exceeds the permitted use, you will need to obtain permission directly from the copyright holder. To view a copy of this license, visit http://creativecommons.org/licenses/by/4.0/.

Reviewers' Comments:

Reviewer #1:
Remarks to the Author:
This paper addresses an important mystery in the field of local translation – why across so many studies are ribosomal protein mRNA detected in distal compartments? This contradicts dogma, where ribosomes are mostly assembled in the nucleolus. This paper attempts to address this question.
Their major claims are 1) There is active translation of new RPs in neurites. 2) 12 new RPs exchange into mature pre-existing ribosomes. 3) Along with this, there is nucleus independent RP incorporation in isolated processes. 4) Some RPs differ in incorporation by subcellular compartment and physiological state.
The main data are:
Figure 1/Extended data 1: Is a relentless amount of ISH to show > a dozen RP RNAs in neurites in hippocampus and in cultured neurons. Positive controls are Camk2a, and a nice negative control is His3.
Figure 2: Is representation of old RF data to argue new translation in slide of RPs, along with puro/PLA labeling for a large number of RPs in neurites that supports this at least in vitro.
Figure 3: Is SILAC labeling for 1-2 hours, followed by cushioned ribosomes and mass spect to look at new RPs that made it into ribosomes. They show clusters of more rapidly turned over RPs, and those that are slower. This is supported by a 3H chase experiment, where the slow clusters tend to continue to increase, while the rapid ones may actually decrease a bit – consistent with very fast turn over (this includes RPL10 and other RPs that the Barna lab had shown are sub stoichiometric)
Figure 4: Shows some RPs can incorporate when nuclear export is blocked by 48 hour of LMB, again by SILAC. It takes some time to get this figure, but it starts with scatterplots (C) which show LMB decreasing new protein incorporation into ribosomes overall. However, in Figure D, once you normalize that, there is a subset that are higher in new protein levels than most others in LMB – these they assume are exchanging.
Figure 5: Is a very similar SILAC experiment, but not adding a perturbation – H202 exposure – that further leads to increased incorporation of 4-5 of the exchanging RPs into ribosomes, suggesting this could be a dynamic and/or repair response.
Overall, I think the data are consistent with their claims, and this is a very interesting work and should be impactful. There are some experiments that are more informative than others, but on the whole I think this should be suitable for publication with minor modifications, perhaps 1 experiment on mRNA half-lives to help interpret data in Figure 4, and some discussion of the limitations of a couple experiments.
Moderate
1) Figure 4, generally: LMB is certainly an imperfect tool for what they want to do. They would like to block ribosome biogenesis, but instead they can block nuclear export. They present evidence that this does indeed block the ability to form new ribosomes (e.g, 5B). And the data they then show that 12 RPs alter their relative abundance of new proteins on old ribosomes supports their main conclusions. (Though they might want to add the word 'normalized' to the Y-axis label of 4d to make it easier to get at a glance).
But LMB is not quite a perfect drug for this: 48 hours of LMB would presumably block mRNA export as well for two days? Over time, this should lead to a depletion of mRNA from the pool available for translation (including RP mRNAs). Could the relative changes across these 12 RPs after LMB treatment simply reflect a different mRNA half-lives among them, with that difference in mRNA abundance leading to the range of differences seen in new protein over time? Are there measures in the literature for the half-lives of these mRNAs (or, if not, can they assess it in their neurons with click-it, similar to how they approach the rRNA) to rule out this alternate explanation for why certain subsets of RPs show more differences than others?
2) The claim of active translation of new RPs in neurites is well supported in culture, in Figure 2, but not as strongly supported in vivo where the presented neuropil RF data would also be expected to contain glial somas that also make ribosomes, and where PLA is not conducted (and indeed, may not be possible). As I can't think of an experiment to address this, this probably warrants a bit of

discussion of the limitations in the discussion section, as the in vitro cultures might be expected to be more dynamic than the slices.

3) I understand why the cytoplasmically assembled RPs might show exchange in the LMB condition, but I am a bit confused by the subset of RPs that are thought to be added in the nucleus during maturation that show up as exchanging. Their data suggests that either LMB blockage of export is incomplete, or that these are not really only assembled in the nucleus. They should perhaps discuss these implications more.

4) The neuropil in figure 1B that is used from RNAseq (from ref 25) also will contain glia. How did they normalize for that here? Same for 2a. On the whole this is a minor concern, as the FISH and PLA provide additional support for their conclusions. But the should probably mention this limitation of these analyses.

Minor
5) Line 111: seems like 27/29 were significantly different from H3. Not all 29 as stated.
6) Line 121: contrary to their statement here, it seems like there are a couple of RPs where the authors do not detect footprints across the whole of the coding sequence in the extended data figure. Is this real? If so it is strange. Though perhaps it might be just parts of the CDS where short fragments could not be readily mapped (repetitive seqs?). If they are unalignable repetitive regions, that could be indicated in the cartoon of the CDS by a difference of color or something, or perhaps this warrants some discussion.
7) Line 268: I am not sure their data fit the phrase 'decidedly extreme halflives,' especially for 10C, where they are sort of in the low middle of the pack for the Y axis.
8) Figure 4B: how is nascent rRNA labeled here? This experiment is not well explained without a thorough read of the methods. It probably warrants also having a line of explanation in the results to clarify what it is showing. It is clear they are blocking nuclear export, but without this panel it is less clear the authors are inhibiting biogenesis of new ribosomes, so I would expand the writing here a bit.
9) Line 75: The results section is missing a header.
10) Figure 5 is interesting. Barna lab had some data on differential mRNA binding by heterogenous ribosomes that contained or were missing particular RPs. Do any of those line up with the H202 responsive RPS here? If so, it might be interesting to look in the Barna data to see if the GO results for mRNA enriched by those RPs might fit with the authors speculation that RP exchange could be related to modulation of a stress response.

Reviewer #2:
Remarks to the Author:
The manuscript of Fusco et al. "The dynamic non-canonical incorporation of ribosomal proteins into neuronal ribosomes" reports that mRNA encoding ribosomal proteins (RPs) are translated in neuropil, incorporate into ribosomes in the cytoplasm and the set of incorporated RPs is regulated by location (cell bodies vs. neurites) and oxidative stress.
1) My main concerns is that many of these findings are not novel. Examples of prior studies that report similar findings are given below. The authors fail to cite them at the relevant parts of the manuscript, when explaining their motivation for a similar experiment. Some of these studies are cited as an afterthought in the discussion part. The effect of oxidative stress on selective incorporation of RPs is interesting, but unfortunately it comes in the last figure as a descriptive piece and no explanation is provided for the biological role of incorporating specifically these 4 RPs under stress.
- On-Site Ribosome Remodeling by Locally Synthesized Ribosomal Proteins in Axons (Shigeoka 2019): this study has the most overlap in terms of findings with the submitted manuscript. It shows that locally synthesized RPs can be incorporated in axonal ribosomes, using metabolic labelling of newly synthesized proteins with AHA (FUNCAT) & incorporation into ribosomes with proximity ligation assay (rRNA & biotin antibodies); it also uses labelling of ribosomes with Cy5-UTP and using FRAP to detect

co-localization of locally synthesized Venus-RPs and Cy5-ribosomes
- Conservation of a core neurite transcriptome across neuronal types and species (von Kügelgen 2020): does analysis of multiple localization and translation datasets to show consistent localization and translation of RP mRNAs in neuronal processes
- Transcriptomic Analysis of Ribosome-Bound mRNA in Cortical Neurites In Vivo (Ouwenaga 2017): combines synaptoneurosomal fractionation with translating ribosome affinity purification to identify ribosome-bound mRNA in neuronal processes
- Dynamic Axonal Translation in Developing and Mature Visual Circuits (Shigeoka 2016): identifies ribosome-bound mRNAs in the retinal ganglion cell axons with axon-TRAP-RiboTag
- Functionally diverse dendritic mRNAs rapidly associate with ribosomes following a novel experience (Ainsley 2014): identifies ribosome-bound mRNAs from the dendrites of CA1 pyramidal neurons in mouse hippocampus
2) My second concern is technical:
- Figure 4 (metabolic labeling combined with isolation of neuronal processes): metabolic labelling is done for 48 hr and is combined with Leptomycin B treatment to inhibit ribosome export from the nucleus. However, Leptomycin B would not prevent transport of proteins and ribosomes between cell bodies and neurites during these two days, so the authors cannot conclude about location-specific incorporation of RPs into ribosomes.
- Figure3,4 & S6a (pSILAC to evaluate incorporation rate of newly synthesized RPs into ribosomes): the authors label newly synthesized RPs with heavy isotopes, then purify ribosomes using sucrose cushion and evaluate incorporation of heavy RPs into ribosomes. The incorporated heavy RPs are estimated as a relatively low fraction of purified ribosomes (~0.004-0.015), and it is unclear how much of this is a background coming from co-purified complexes, supernatant carryover etc. Indeed, Figure S6a shows that even after treatment with EDTA, which should have disrupted 80S monosomes, RPs can still be detected in the cushion fraction. The authors should evaluate the background, for example by using cushion fraction after treatment with EDTA as a control. Introducing medium isotopes would allow to do the analysis of plus/minus EDTA cushion samples together as it is usually done for SILAC: heavy without EDTA/medium with EDTA and a label swap.

Reviewer #3:
Remarks to the Author:
The manuscript by Fusco and co-workers uses a combination of different methods (RNAseq, ribosome profiling, proteomics, imaging) to study incorporation of ribosomal proteins into already existing ribosomes in neuronal cells. They provide evidence that ribosomal proteins are translated and locally incorporated into dendrites and axons. They conclude that there is an alternative (”non-canonical”) way how ribosomal proteins are incorporated into ribosomes that is different from canonical ribosome assembly.

I think this is a very nice paper that convincingly shows that ribosomes in neurons are more dynamic than initially thought. A particular strength is the use of orthogonal methods that all provide independent evidence. While the biological significance of the findings remain to be investigated, I think the observation of non-canonical incorporation of specific ribosomal protein is in itself an exciting observation that deserved publication in Nature Communication. The paper is also very well and carefully written. However, there are a number of points that I think should be adressed before it can be formally accepted:

1. Fig. 1C: The detection of one proteasomal mRNA is quite intriguing. Can the authors say a little more about this, even though it is not related to the main story of this paper?
2. Rps30-FAU is an interesting case because this protein is posttranslationally processed. Is there any evidence for this occurring in the neutropil?
3. Fig. 1F: Which sample was used as the single control in the Dunnetts Test? This info should be added to the legend.

4. Extended figure 1b: In this figure a wilcoxon test was used rather than Anova followed by Dunnets. I do not understand the reason for this difference. I think the Anova+Dunetts procedure is better since it efficiently eliminates false positives arising from multiple hypothesis testing.
5. To assess whether the ribosome profiling data reflects genuine translation, it would be informative to also analyze the periodicity of the reads. I am missing an analysis of this kind.
6. Line 150: The authors did not use pSILAC (as in Schwanhausser, Proteomics, 2009 or Selbach, Nature, 2008) but rather dynamic SILAC (as in Doherty, JPR, 2009 and Schwanhausser, Nature, 2011). The nomenclature should be adjusted throughout the paper.
7. Line 203: "Importantly, we note that the 12/12 mRNAs of the rapidly incorporating RPs were detected in the neuropil RNA-seq dataset (Fig. 1c), and 11/12 were detected in the ribosome footprints dataset (Fig. 2a and Extended Data 3)." I am wondering about "false negatives". In other words, how many ribosomal mRNAs are enriched in the RNAseq/footprint data although the corresponding proteins do not show a rapid exchange? Is the overlap actually significant? A hypergeometric test could answer this question. And how do the authors interpret the enriched mRNAs encoding slowly exchanged ribosomal proteins?
8. Fig 4C: It would help interpretation to use the same scale on the x and y axis.
9. How do the authors interpret the inconsistent behaviour of Rplp0 in fig. 3b and 4d?
10. Line 265 "In this regard, we noted a significant negative correlation between the level of incorporation of an RP and its half-life in both cultured neurons and intact brain, with exchanging RPs showing both the shortest and decidedly extreme half-lives when compared to other RPs (Extended Data Fig. 10b-c, 36,37)" In this context, it would be useful to mention that ribosomal proteins tend to be produced in superstoichiometric quantities relative to rRNAs, which results in rapid degradation of a significant fraction of the newly synthesized pool that cannot be incorporated into ribosomes (McShane et al., Cell, 2016). Hence, the shorter half-life of the exchanging RPs might reflect their higher degradation rate in their unassembled monomeric state.
11. Ext Fig 10D: It is not clear to me how exactly the "normalised abundances" where calculated. The legend says these are "protein levels normalized to the median of the corresponding ribosomal subunit". Are these iBAQ values? If not, how did the authors obtain absolute abundance estimates for the different RPs?

Matthias Selbach

We thank the reviewers for their valuable time and for the critiques and suggestions. Below we have excerpted the Reviewer summary comments and their individual points and our responses.

REVIEWER COMMENTS

Reviewer #1 (Remarks to the Author):

This paper addresses an important mystery in the field of local translation – why across so many studies are ribosomal protein mRNA detected in distal compartments? This contradicts dogma, where ribosomes are mostly assembled in the nucleolus. This paper attempts to address this question.
Their major claims are 1) There is active translation of new RPs in neurites. 2) 12 new RPs exchange into mature pre-existing ribosomes. 3) Along with this, there is nucleus independent RP incorporation in isolated processes. 4) Some RPs differ in incorporation by subcellular compartment and physiological state.

……..

Overall, I think the data are consistent with their claims, and this is a very interesting work and should be impactful. There are some experiments that are more informative than others, but on the whole I think this should be suitable for publication with minor modifications, perhaps 1 experiment on mRNA half-lives to help interpret data in Figure 4, and some discussion of the limitations of a couple experiments.

1) Figure 4, generally: LMB is certainly an imperfect tool for what they want to do. They would like to block ribosome biogenesis, but instead they can block nuclear export. They present evidence that this does indeed block the ability to form new ribosomes (e.g, 5B). And the data they then show that 12 RPs alter their relative abundance of new proteins on old ribosomes supports their main conclusions. (Though they might want to add the word 'normalized' to the Y-axis label of 4d to make it easier to get at a glance). But LMB is not quite a perfect drug for this: 48 hours of LMB would presumably block mRNA export as well for two days? Over time, this should lead to a depletion of mRNA from the pool available for translation (including RP mRNAs). Could the relative changes across these 12 RPs after LMB treatment simply reflect a different mRNA half-lives among them, with that difference in mRNA abundance leading to the range of differences seen in new protein over time? Are there measures in the literature for the half-lives of these mRNAs (or, if not, can they assess it in their neurons with click-it, similar to how they approach the rRNA) to rule out this alternate explanation for why certain subsets of RPs show more differences than others?

The reviewer is concerned that the differential effects of LMB treatment on RP incorporation could be due to differential depletion of RP mRNAs, owing to a potential difference in half-life. A recent publication from our lab measured the half-lives of individual mRNAs in cultured neurons (Tushev et al. 2018). To address the reviewer's question, we used this dataset to examine whether the mRNAs encoding for the 12 exchanging RPs have a longer half-life than the other RPs, to potentially explain their differential incorporation after LMB treatment. We observed no difference between the two groups (see figure below). We have now added this figure to supplementary figure 11a of the revised manuscript. Moreover, we point out that these 12 RPs are also seen as exchangers in figure 3 (without LMB treatment and suppl.fig. 11b).

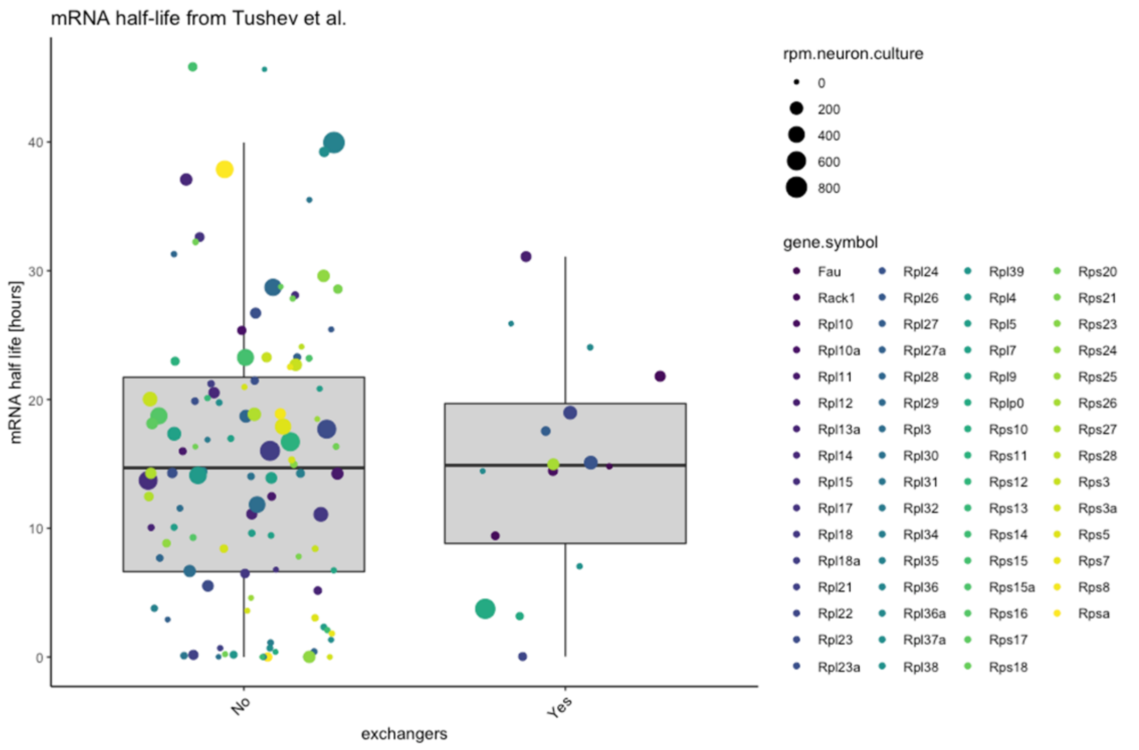

Data sourced from Tushev et al. 2018. Transcript isoforms of the same gene are shown in the same color. The size of each dot is proportional to the transcript expression level in cultured neurons (measured as rpm, reads per million).

2) The claim of active translation of new RPs in neurites is well supported in culture, in Figure 2, but not as strongly supported in vivo where the presented neuropil RF data would also be expected to contain glial somas that also make ribosomes, and where PLA is not conducted (and indeed, may not be possible). As I can't think of an experiment to address this, this probably warrants a bit of discussion of the limitations in the discussion section, as the in vitro cultures might be expected to be more dynamic than the slices.

We have now edited the text in the results section after figure 2a acknowledging the presence of glia cells in the sequenced material.

3) I understand why the cytoplasmically assembled RPs might show exchange in the LMB condition, but I am a bit confused by the subset of RPs that are thought to be added in the nucleus during maturation that show up as exchanging. Their data suggests that either LMB blockage of export is incomplete, or that these are not really only assembled in the nucleus. They should perhaps discuss these implications more.

Our current understanding of the order and location of RP assembly during ribosome biogenesis is often derived from indirect evidence. For example, inferences about a given RP's assembly in the nucleolus, nucleus or cytoplasm are typically derived from co-purification experiments with ribosome-binding protein or pre-RNA species, or, according to which pre-RNA intermediates are still generated after a given RP is knocked down. Indeed, recent studies (Ohmayer et al. 2013, Gamalinda et al. 2014, Ferreira-Cerca et al. 2005) suggest that some of our putative exchangers (Rps30, Rpl22, Rpl27, Rpl38, Rpl12, Rpl36) are incorporated during canonical ribosome biogenesis in the nucleus. However, these

studies cannot exclude the possibility that these RPs might also be assembled into (pre-existing) ribosomes in the cytosol. We therefore are open to the possibility that an RP might exchange in a manner that is inconsistent with our current understanding of the order of incorporation during canonical ribosome biogenesis. We believe that a better predictor of a RP's capacity to exchange is its half-life and its position (more on the surface vs. buried) in the ribosome 3D structure.

4) The neuropil in figure 1B that is used from RNAseq (from ref 25) also will contain glia. How did they normalize for that here? Same for 2a. On the whole this is a minor concern, as the FISH and PLA provide additional support for their conclusions. But the should probably mention this limitation of these analyses.

These data are not normalized and glia could still contribute to the signal. That's why we corroborate the evidence with FISH and Puro-PLA. We have now edited the revised text to acknowledge the presence of glia in the sequenced material.

Minor:

5) Line 111: seems like 27/29 were significantly different from H3. Not all 29 as stated.

We fixed this text.

6) Line 121: contrary to their statement here, it seems like there are a couple of RPs where the authors do not detect footprints across the whole of the coding sequence in the extended data figure. Is this real? If so it is strange. Though perhaps it might be just parts of the CDS where short fragments could not be readily mapped (repetitive seqs?). If they are unalignable repetitive regions, that could be indicated in the cartoon of the CDS by a difference of color or something, or perhaps this warrants some discussion.

Indeed, for some genes (e.g. Rps9, Rpl37a, Rpl21) we were not able to detect reads continuously over the entire coding sequence. We believe this is due to a sensitivity issue, in fact for those genes the number of reads is overall very low. However, as this applies both to the somas and dendrites, we think the data still supports the interpretation, that these mRNA are translated in the neuropil. Nevertheless, we removed "entire" from the text to avoid confusion.

7) Line 268: I am not sure their data fit the phrase 'decidedly extreme halflives,' especially for 10C, where they are sort of in the low middle of the pack for the Y axis.

We have edited the text.

8) Figure 4B: how is nascent rRNA labeled here? This experiment is not well explained without a thorough read of the methods. It probably warrants also having a line of explanation in the results to clarify what it is showing. It is clear they are blocking nuclear export, but without this panel it is less clear the authors are inhibiting biogenesis of new ribosomes, so I would expand the writing here a bit.

We edited the text in the results section to explain this better.

9) Line 75: The results section is missing a header.

Fixed.

10) Figure 5 is interesting. Barna lab had some data on differential mRNA binding by heterogenous ribosomes that contained or were missing particular RPs. Do any of those line up with the H202 responsive RPS here? If so, it might be interesting to look in the Barna data to see if the GO results for mRNA enriched by those RPs might fit with the authors speculation that RP exchange could be related to modulation of a stress response.

Unfortunately, none of the candidates we see differentially exchange after oxidative stress are on the list of RPs the Barna lab performed Ribo-seq for (RPS25- and RPL10a-containing ribosomes).

Reviewer #2 (Remarks to the Author):

The manuscript of Fusco et al. “The dynamic non-canonical incorporation of ribosomal proteins into neuronal ribosomes” reports that mRNA encoding ribosomal proteins (RPs) are translated in neuropil, incorporate into ribosomes in the cytoplasm and the set of incorporated RPs is regulated by location (cell bodies vs. neurites) and oxidative stress.

1) My main concerns is that many of these findings are not novel. Examples of prior studies that report similar findings are given below. The authors fail to cite them at the relevant parts of the manuscript, when explaining their motivation for a similar experiment. Some of these studies are cited as an afterthought in the discussion part. The effect of oxidative stress on selective incorporation of RPs is interesting, but unfortunately it comes in the last figure as a descriptive piece and no explanation is provided for the biological role of incorporating specifically these 4 RPs under stress.
- On-Site Ribosome Remodeling by Locally Synthesized Ribosomal Proteins in Axons (Shigeoka 2019): this study has the most overlap in terms of findings with the submitted manuscript. It shows that locally synthesized RPs can be incorporated in axonal ribosomes, using metabolic labelling of newly synthesized proteins with AHA (FUNCAT) & incorporation into ribosomes with proximity ligation assay (rRNA & biotin antibodies); it also uses labelling of ribosomes with Cy5-UTP and using FRAP to detect co-localization of locally synthesized Venus-RPs and Cy5-ribosomes
- Conservation of a core neurite transcriptome across neuronal types and species (von Kügelgen 2020): does analysis of multiple localization and translation datasets to show consistent localization and translation of RP mRNAs in neuronal processes
- Transcriptomic Analysis of Ribosome-Bound mRNA in Cortical Neurites In Vivo (Ouwenaga 2017): combines synaptoneurosomal fractionation with translating ribosome affinity purification to identify ribosome-bound mRNA in neuronal processes
- Dynamic Axonal Translation in Developing and Mature Visual Circuits (Shigeoka 2016): identifies ribosome-bound mRNAs in the retinal ganglion cell axons with axon-TRAP-RiboTag
- Functionally diverse dendritic mRNAs rapidly associate with ribosomes following a novel experience (Ainsley 2014): identifies ribosome-bound mRNAs from the dendrites of CA1 pyramidal neurons in mouse hippocampus
We are disappointed that the reviewer does not see the novelty of our findings. While several groups (cited in our paper) have detected RP transcript localization and translation, there is only one other group that has reported the cytosolic incorporation of locally synthesized RPs. As cited in our paper, Shigeoka et al studied axonal ribosomes in retinal ganglion neurons of Xenopus. We studied dendritic and axonal ribosomes in rodent hippocampal neurons- arguably the most studied cell-type in the brain. Axons and dendrites

are different compartments of neurons- endowed with very different information processing capabilities. It should not be assumed that the cell biology of the axon is the same as the dendrite. Although some experiments of ours and others do not differentiate (e.g. studies of "neurites" in compartmentalized chambers) between these two compartments, one of the strengths of our study is the identification of RP mRNAs and translation products dendritic compartment, where many studies of local translation have been conducted. We have included mention of the Shigeoka et al 2019 now also in the introduction of our paper. We believe that our mass spectrometry data, in particular, are also a big strength of the present paper because we include a "no heavy" control and we also have strong evidence that we detected the expected exchanging proteins (e.g. Rack1, Rplp1, Rplp2) which were not detected in Shigeoka et al. We have cited all of the above papers in in our manuscript except Ainsley, which was critically flawed and lacked a control group. This paper could not be cited without several lines of accompanying text to indicate the flaws.

2) My second concern is technical:
- Figure 4 (metabolic labeling combined with isolation of neuronal processes): metabolic labelling is done for 48 hr and is combined with Leptomycin B treatment to inhibit ribosome export from the nucleus. However, Leptomycin B would not prevent transport of proteins and ribosomes between cell bodies and neurites during these two days, so the authors cannot conclude about location-specific incorporation of RPs into ribosomes.

The reviewer is correct that we cannot unambiguously assert that the incorporation occurs locally, however the fact that some new RPs show either differential levels between compartments or even higher levels in ribosomes purified from neurites, strongly suggests that at least the recruitment of these proteins is location-specific. We have adjusted our revised text to reflect these ideas.

- Figure3,4 & S6a (pSILAC to evaluate incorporation rate of newly synthesized RPs into ribosomes): the authors label newly synthesized RPs with heavy isotopes, then purify ribosomes using sucrose cushion and evaluate incorporation of heavy RPs into ribosomes. The incorporated heavy RPs are estimated as a relatively low fraction of purified ribosomes (~0.004-0.015), and it is unclear how much of this is a background coming from co-purified complexes, supernatant carryover etc. Indeed, Figure S6a shows that even after treatment with EDTA, which should have disrupted 80S monosomes, RPs can still be detected in the cushion fraction. The authors should evaluate the background, for example by using cushion fraction after treatment with EDTA as a control. Introducing medium isotopes would allow to do the analysis of plus/minus EDTA cushion samples together as it is usually done for SILAC: heavy without EDTA/medium with EDTA and a label swap.

We reproduced by Mass Spectrometry the de-enrichment of RPs (via Western blot) when the cushion is performed in the presence of EDTA (see figure below, panel ***a****). To unambiguously test whether the low heavy signal that we detected after short SILAC labeling comes from non-specific background, we labeled neurons with heavy amino acids for 2 hours (or not, for the negative control) and performed a cushion in the presence or absence of EDTA. Indeed, the heavy signal was drastically reduced in the presence of EDTA (see figure below, panel* ***b****), confirming the specificity of our labeling for new assembled ribosomes. In fact, the reduction with EDTA of the heavy signal is not significantly different from the reduction of the total signal, for both exchanging (in purple) and non-exchanging RPs (in black). Altogether, this data shows that the detection of heavy signal comes indeed from assembled ribosomes, and not from non-specific background. We thank the reviewer for suggesting this experiment, which has corroborated the specificity of our paradigm. We have now included this data in the revised manuscript (Supplementary figure 6e-g).*

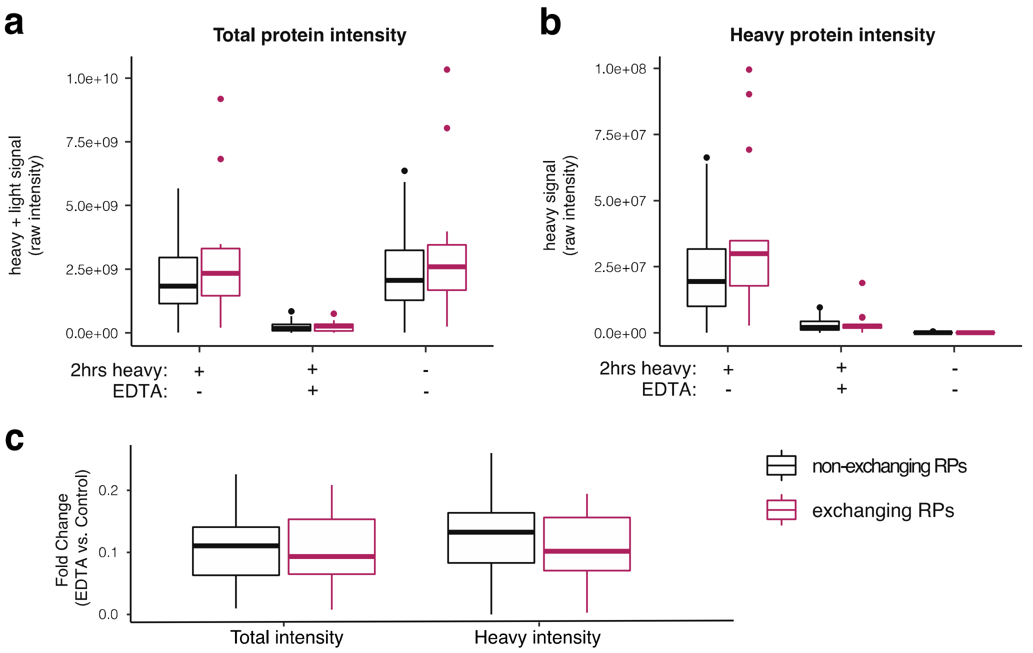

Reviewer #3 (Remarks to the Author):

The manuscript by Fusco and co-workers uses a combination of different methods (RNAseq, ribosome profiling, proteomics, imaging) to study incorporation of ribosomal proteins into already existing ribosomes in neuronal cells. They provide evidence that ribosomal proteins are translated and locally incorporated into dendrites and axons. They conclude that there is an alternative ("non-canonical") way how ribosomal proteins are incorporated into ribosomes that is different from canonical ribosome assembly.

I think this is a very nice paper that convincingly shows that ribosomes in neurons are more dynamic than initially thought. A particular strength is the use of orthogonal methods that all provide independent evidence. While the biological significance of the findings remain to be investigated, I think the observation of non-canonical incorporation of specific ribosomal protein is in itself an exciting observation that deserved publication in Nature Communication. The paper is also very well and carefully written. However, there are a number of points that I think should be adressed before it can be formally accepted:

1. Fig. 1C: The detection of one proteasomal mRNA is quite intriguing. Can the authors say a little more about this, even though it is not related to the main story of this paper?
Psmb9 and Psmb8 show the highest and second highest fold change in the neuropil, respectively. This heterogeneity within the proteasome is indeed very interesting, and another project in the lab is working on it. Please see also Perez et al., eLIFE, 2021 for more data on proteasome mRNAs in individual dendrites.

2. Rps30-FAU is an interesting case because this protein is posttranslationally processed. Is there any evidence for this occurring in the neutropil?

We were also very much intrigued by the possibility of local post-translational processing of Rps30-fau. Unfortunately, the proteins involved in this process have not been fully characterized yet. A very recent pre-print has suggested the deubiquitinase USP36 might be responsible for the FAU cleavage in HeLa cells (van den Heuvel 2021 biorxiv). Unfortunately, we couldn't identify the predicted rat homolog of this protein (D3ZNQ4_RAT)

in our proteomics data sets (neither in the whole neuronal lysate used in the present study, nor in neuropil or somata enriched lysates from Biever et al. 2020).

3. Fig. 1F: Which sample was used as the single control in the Dunnetts Test? This info should be added to the legend.

His3 was used as control in the Dunnett's test. Legend has been edited accordingly.

4. Extended figure 1b: In this figure a wilcoxon test was used rather than Anova followed by Dunnets. I do not understand the reason for this difference. I think the Anova+Dunetts procedure is better since it efficiently eliminates false positives arising from multiple hypothesis testing.

We thank the reviewer for noticing this. It was a mistake in the legend. Indeed, the test performed was a Anova+Dunett's. We corrected the legend.

5. To assess whether the ribosome profiling data reflects genuine translation, it would be informative to also analyze the periodicity of the reads. I am missing an analysis of this kind.

For this analysis, we refer to the original paper Biever et al. 2020, where the periodicity of the reads was confirmed for both the neuropil and somata ribosome profiling data. We now mention the 3 nucleotide periodicity in figure legend of the revised manuscript.

6. Line 150: The authors did not use pSILAC (as in Schwanhausser, Proteomics, 2009 or Selbach, Nature, 2008) but rather dynamic SILAC (as in Doherty, JPR, 2009 and Schwanhausser, Nature, 2011). The nomenclature should be adjusted throughout the paper.

Thanks for the correction. We have changed the nomenclature throughout the revised manuscript.

7. Line 203: "Importantly, we note that the 12/12 mRNAs of the rapidly incorporating RPs were detected in the neuropil RNA-seq dataset (Fig. 1c), and 11/12 were detected in the ribosome footprints dataset (Fig. 2a and Extended Data 3)." I am wondering about "false negatives". In other words, how many ribosomal mRNAs are enriched in the RNAseq/footprint data although the corresponding proteins do not show a rapid exchange? Is the overlap actually significant? A hypergeometric test could answer this question. And how do the authors interpret the enriched mRNAs encoding slowly exchanged ribosomal proteins?

As we mention in the discussion section, it is interesting to note that "only" 12 RP show dynamic incorporation behavior, while we and others detected the mRNA of almost all of them in neuronal processes. This might be due to:

- *Technical limitation: relative (and not absolute) analysis among the 80 RPs allows us to identify group of proteins with more dynamic behavior than the others, but we cannot exclude the possibility that the less dynamic RPs might also be able to exchange.*

- *Biological issue: in different physiological conditions, the incorporation probability of different RPs might change, leading to a different "identity" of exchanging RPs.*

8. Fig 4C: It would help interpretation to use the same scale on the x and y axis.

Done.

9. How do the authors interpret the inconsistent behaviour of Rplp0 in fig. 3b and 4d?

Rplp0 comes up in the rapid and dynamic incorporation group in fig.3b (cluster C) and in the biogenesis-independent incorporation group in fig.4d. These observations are consistent with our interpretation of Rplp0 possessing the capacity to exchange.

10. Line 265 "In this regard, we noted a significant negative correlation between the level of incorporation of an RP and its half-life in both cultured neurons and intact brain, with exchanging RPs showing both the shortest and decidedly extreme half-lives when compared to other RPs (Supplementary Fig. 10b-c, 36,37)" In this context, it would be useful to mention that ribosomal proteins tend to be produced in superstoichiometric quantities relative to rRNAs, which results in rapid degradation of a significant fraction of the newly synthesized pool that cannot be incorporated into ribosomes (McShane et al., Cell, 2016). Hence, the shorter half-life of the exchanging RPs might reflect their higher degradation rate in their unassembled monomeric state.

True. Thanks for pointing this out. The revised text now includes a sentence on it.

11. Ext Fig 10D: It is not clear to me how exactly the "normalised abundances" where calculated. The legend says these are "protein levels normalized to the median of the corresponding ribosomal subunit". Are these iBAQ values? If not, how did the authors obtain absolute abundance estimates for the different RPs?

Protein levels correspond to SILAC ratio (over internal standard) as measured in the original paper (Imami et al. 2018). In the method sections we explain in more details how the normalized abundances are calculated: "The abundance of ribosomal proteins in selected fractions (40S, 60S, 80S and polysome) were extracted and the value of each ribosomal protein was normalized over the median abundance of all proteins of the corresponding subunit within each fraction (when considering proteins of the small subunit, the fraction corresponding to the 60S was excluded, and vice versa for the large subunit and the 40S fraction)." We have now hopefully made this clearer in the figure legend as we refer to the methods section.

Matthias Selbach

Reviewers' Comments:

Reviewer #1:
Remarks to the Author:
The authors have addressed my concerns. It is a very nice manuscript.

Reviewer #3:
Remarks to the Author:
The authors addressed all points I raised. I think this interesting paper can now be accepted for publication.
